# Supplementary material for: Widespread occurrence and relevance of phosphate storage in foraminifera
Source: Nature. 2025 Jan 15;638(8052):1000–6. doi: 10.1038/s41586-024-08431-8 (PMC11864969; doi:10.1038/s41586-024-08431-8)
Supplement: Supplementary file 1 — This file contains Supplementary Notes, Figs. 1–6, Tables 1–7 and references. [file 41586_2024_8431_MOESM1_ESM.pdf]

nature portfolio

https://doi.org/10.1038/s41586-024-08431-8

Supplementary information

Widespread occurrence and relevance of phosphate storage in foraminifera

In the format provided by the authors and unedited

Supplementary Information for

"Widespread occurrence and relevance of phosphate storage in foraminifera"

by N. Glock *et al.*

Supplementary notes

SN1: ^{31}P-NMR Spectroscopy

^{31}P-NMR spectra of the P-compounds that have been extracted from replicates of samples containing ~1000 living specimens of *Ammonia confertitesta* show reproducable discrete peaks (Fig.SF1 A&B). ATP was recognized, due to its typical triple peak structure [1]. The α-P peak appeared at -10.40 ppm, the β-P peak at -20.43 ppm and the γ-P peak at -4.88 ppm. The β- and γ-P peaks are superimposed by smaller peaks that likely correspond to traces of other polyphosphates. Pyrophosphate was recognized by spiking the sample with pyrophosphate (Fig.SF2). The peak at 0 ppm was inconclusive (marked with a * in Fig.SF1). It is very similar to creatine phosphate [1]. However, after spiking the sample with creatine phosphate, the peak was observed at a slightly higher chemical shift of about 0.77 ppm (Fig.SF2).One reason could have been a slight change in the pH value over time. One peak at +1.05 ppm could not be identified, while the peaks at +4.14, 4.98 and 5.50 ppm correspond likely to AMP and orthophosphate. The spectrum of the *in vivo* measurement on the living *A. confertitesta* (Fig.SF1C) had much broader lines, than the spectra of the extracted P-compounds. However, the maxima correspond with the position of lines in the extracted samples. Severe line broadening was expected due to the inhomogeneity of the sample containing the living individuals and due to a drastic change in the diffusion correlation times within the cells, leading to much shorter transversal (T_2) relaxation times.

SN2: Additional Cryo-SEM images

As discussed in the main manuscript, the circular structures of 0.5 – 3 µm diameter were frequently observed in the cytoplasm of several cryo-fixed *A. venata* specimens (examples in fig.SF3). The P-rich structures in *B. spissa* that are more concentrated in P, compared to *A. venata*, look different. The structures in *B. spissa* have a grape like appearance and look similar as other Ca-polyphosphate microparticles, that are known to store metabolic energy [2,3].

SN3: Additional TEM images of *Ammonia venata* thin sections and related EDS-spectra

As discussed in the main manuscript, TEM images of *A. venata* thin sections are lacking the frequent circular structures with 0.5 - 3 µm diameter, that are observed under cryo-SEM after cryo-fixation (see fig.3 and Extended Data Fig.E1). Instead, abundant empty vesicles of similar size and shape have been observed on the thin section. This has been interpreted as an artifact, due to the sample fixation and preparation technique for TEM thin section, as discussed in the main manuscript. We hypotesized that the structures lost their content during TEM-preparation. This is additionally supported by the fact, that we observed phosphorus hotspots in the cytoplasm of *A. venata*, using EDS on the cryo-fixed specimens (Fig.3) but not on the TEM thin sections. In total 40 of 41 point measurements with EDS on the thin sections showed no phosphorus signal above the background noise level (for examples see Extended Data Fig.E3&E4). Only one spot had a visible phosphorus signal (Extended Data Fig.E4B2). This was a vesicle of similar size and shape that still had visible content (Extended Data Fig.E4B).

SN4: Comparative genomics and metabarcoding results

<u>Comparative genomics:</u> Initially, predicted proteins of *R. filosa* were used as input for KAAS using the bi-directional best blast approach, which identified the protein accession ETO25900.1 as creatine kinase homolog. Further homologs were searched in public transcriptomes sequences for *Ammonia confertitesta* (GIDR00000000.1) and *Globobulimina pacifica* (GIHI00000000.1) as well as in a new transcriptome assembly produced for *Ammonia veneta*

(SRA: SRR18700766). The new assembly was produced based on sequencing reads from SRA using the Trinity assembly tool[4] (v2.15.1, Parameters: '--trimmomatic --normalize_max_read_cov 20'). The transcriptomes were prefiltered before processing via KAAS server to reduce the number of input sequences. Here similarity searches via NCBI blast[5] (tblastn, 2.12.0+) were applied with ETO25900.1 and a known human creatine kinase (GeneID hsa:1152) as queries against the transcriptome assembly sequences. Nucleotide sequences of all resulting blast hits were used as input for KAAS in single-directional best hit mode. Additional create kinase sequences (annotated as K00933) were obtained from the KEGG database. This resulted in multiple candidates assigned to K00933 with accessions GIDR01053249.1 (*A. confertitesta*) and GIHI01127577.1 (*G. pacifica*) being two representative examples. A phylogenetic tree of the Creatine Kinase in the different analyzed foraminifera species is shown in fig. SF4. Here potential kreatine kinase sequences were first translated via getorf tool[6] (EMBOSS:6.6.0.0) and representatives sequences were aligned via MAFFT (v7.490, Parameters: '--maxiterate 1000 –localpair' [7]). The tree was rooted between the taxonomic groups Sar (+ Guillardia theta) and Opisthokonta. Finally, the phylogenetic tree was constructed using IQ-TREE (2.0.7, Parameters: '--alrt 1000 -B 1000',[8]).

Metabarcoding results: All clean reads were combined and assembled with Trinity 2.14.0 [4].The 18S rRNA in *Ammonia* sp. (SRR1300434) were identified by BlastN using partial 18S rRNA of *Ammonia confertitesta* (MK032924) as queries at an e value of $1 \times 10{-}10$. Nucleotide sequences of hitted contig was added to known 18S rRNA alignments [9]. The 18S rRNA dataset was aligned using MAFFT 7.427 [7], and the resulting alignments were curated using trimAl 1.2 [10] with the gappyout option. Maximum likelihood phylogenetic analysis in IQ-TREE 1.6.7 [8] was performed using TIM + R4 [11] as the best fitting model chosen by ModelFinder [12]. Node supports were obtained from ultrafast bootstrap approximation [13] with 1000 replicates and SH-aLRT [14] with 1000 bootstrap replicates. Results are shown in fig. SF5. All the sequence data are available in the NCBI database (https://www.ncbi.nlm.nih.gov/).

SN5: Intracellular phosphate concentrations of all individual measurements

For this study, intracellular phosphate storage has been measured for 33 samples of living benthic foraminifera of 15 different species (Tab.ST.4).

SN6: Population density and total phosphate storage of *A. confertitesta* in the Wadden Sea

The sample that has been taken from the top cm of the sediments in Friedrichskoog (May 2023) to determine the abundance of living benthic foraminifera showed an extremely low biodiversity. The assemblage was completely dominated by *A. confertitesta*, which was the only living species, present at this location during sampling time, except agglutinating foraminifera attached to snails within the size fraction > 2 mm. The population density of living *A. confertitesta* was 417 ind. cm^{-3}. Total phosphate storage in in the top cm of the sediments was calculated, using the average individual phosphate content for *A. confertitesta* (413 pmol ind^{-1}; Tab.1) and the population density.

$$417\ \frac{ind.}{cm^3} \times 413\ \frac{pmol}{ind.} = 172221$$

This corresponds to a total phosphate storage of 1722210000 pmol m^{-2} (or 0.00172 mol m^{-2}). The molar mass for phosphate is 94.97 g mol^{-1}, which corresponds to a total of 0.163 g of phosphate m^{-2} of sediments, stored in *A. confertitesta*. The Wadden Sea covers a total area of

11,500 km^2 (Federal Ministry for the Environment, Nature Conservation, Nuclear Safety and Consumer Protection; Germany [15]). Accordingly, the weight of the total amount of phosphate stored within cells of *A. confertitesta* in the top cm of the sediments of the whole Wadden Sea can be calculated as:

$$0.00172\ \frac{mol}{m^2} \times 94.97\ \frac{g}{mol} \times 11500000000\ m^2 = 187850660\ g$$

This corresponds to a total phosphate storage of ~1880 T within cells of *A. confertitesta* in the top cm of the sediments of the whole Wadden Sea. The yearly consumption of phosphorus containing fertilizers by German agriculture in 2021/2022 was 115,000 T (https://de.statista.com/statistik/studie/id/78379/dokument/mineralische-duengemittel/). The phosphorus content in commercial phosphorus fertilizer is highly variable and can be between 7 – 42 weight% P_2O_5. Thus, the total phosphate storage within cells of *A. confertitesta* in the top cm of the sediments of the whole Wadden Sea during the snapshot of a spring bloom would be ~5% of the yearly P-consumption by commercial fertilizers for German agriculture. Due to the high abundances during sampling in Friedrichskoog, this estimation should be seen as an upper limit.

SN7: Individual results of foraminiferal biovolume determination

Biovolumes of 850 individual benthic foraminifera of 16 different species were determined during this study (Tab.ST5). The biovolumes were used to calculate the intracellular phosphate concentrations (in µM) from the individual intracellular phosphate content (in pmol ind^{-1}).

SN8: Total benthic foraminiferal phosphate storage in the Southern North Sea and off Peru in relation to riverine phosphate runoff

Southern North Sea: The total phosphate storage in foraminifera at the analyzed 135 stations from the North Sea ranged from 0.0000 to 0.1630 g of phosphate m^{-2} of sediments (see suppl. tab.ST6) with an average of 0.0059 g m^{-2} (±0.0014 g m^{-2}; 1SE).The total area in the region of interest of the Southern Nordic Sea covers ~45000 km^2 (suppl. fig.SF6 A). Using the average foraminiferal phosphate storage of 0.0059 g m^{-2}, this results in a total of 265 t of phosphate stored in benthic foraminifera within the region of interest. The total riverine phosphate runoff from the continent to the region of the Southern Nordic Sea in 2019 was 2583 t/yr [16] (see also fig.4). This indicates that benthic foraminifera might buffer ~37 days of riverine phosphate runoff into this region. Since we neglected all foraminifera species with unknown phosphate storage that belong to a genus of which no other species have been analyzed for phosphate storage, yet, and many of the datasets only included the top cm of sediments, which neglects many infaunal species, we consider the total benthic foraminiferal phosphate storage to be even higher.

Peru: The total phosphate storage in foraminifera at the analyzed 35 stations off Peru were generally higher than at the Southern Nordic Sea and ranged from 0.0010 to 0.3306 g of phosphate m^{-2} of sediments (See suppl. tab.ST7) with an average of 0.0315 g m^{-2} (± 0.0101 g m^{-2}; 1SE). The total area in the region of interest of the Southern Nordic Sea covers ~40000 km^2 (suppl. fig.SF6 B). Using the average foraminiferal phosphate storage of 0.0315 g m^{-2}, this results in a total of 1260 t of phosphate stored in benthic foraminifera within the analyzed area. In comparison to the Southern Nordic Sea, data for the total riverine phosphate runoff from this region was not available. Nevertheless, instead of the total phosphate runoff, Riverine total phosphorus runoff to the Peruvian coast from 10 – 15°S has been taken from a global modeling

study for the year 2015[17]. Since “total phosphorus” includes all kinds of inorganic and organic phosphorus compounds and is generally higher than the phosphate runoff. This results in a total riverine phosphorus runoff of 7285 t/yr to the Peruvian coast from 10 - 15°S (see also fig.4 and[17]). Phosphate has a higher molar weight (94.97 g/mol) than phosphorus (30.97 g/mol), which means that the phosphorus has to be converted to phosphate, to be comparable with the intracellular phosphate storage, which results in a total phosphate runoff to the region of 22340 t/yr. This indicates that benthic foraminifera might buffer ~21 days of riverine phosphrous runoff to the Peruvian coast from 10 - 15°S.

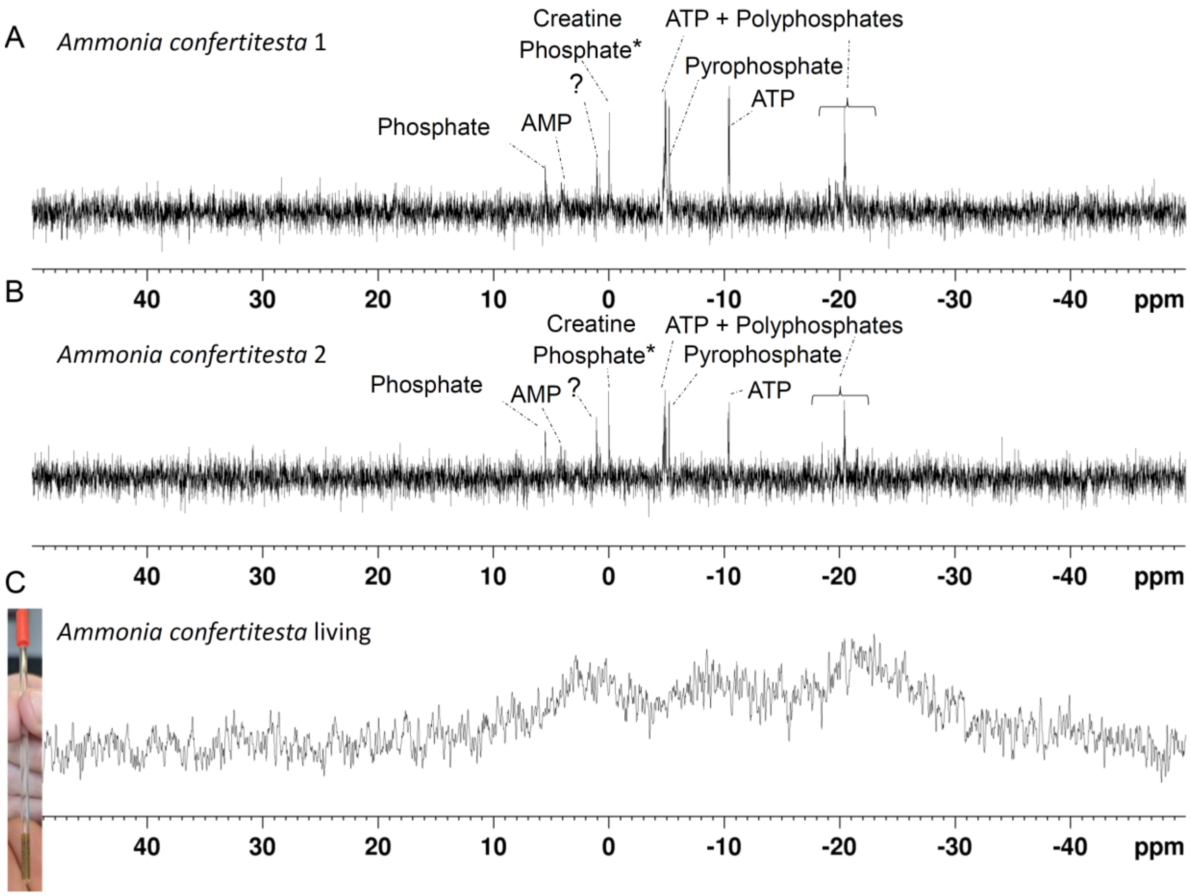

Fig. SF1: ^{31}P-NMR spectra of P-compounds in or extracted from *Ammonia confertitesta*. A&B: Two replicates for spectra of compounds that have been extracted using alkalic lysis (See methods S1.2). Identifiable peaks have been marked. Peaks with "?" have not been identified. The peak marked with "*" has a chemical shift very close to Creatine Phosphate (see fig.SF2) and most likely is Creatine Phosphate. C: ^{31}P-NMR spectrum measured within an NMR-tube filled with 1500 specimens of living *A. confertitesta* (see photo of the living individuals in the NMR tube at the left side of the spectrum). Note that this spectrum is much more complex than A&B.

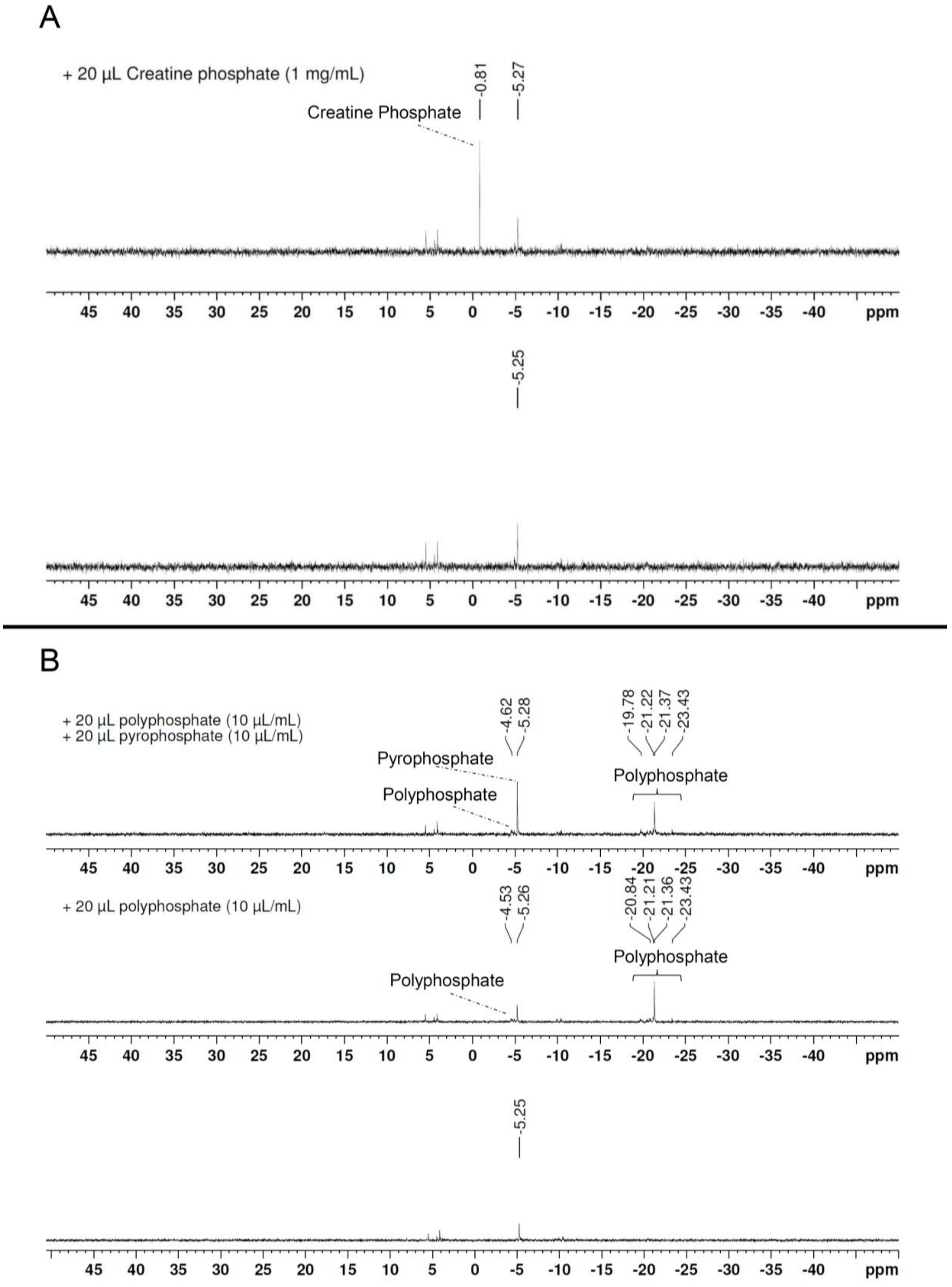

Fig. SF2: Refence spectra of (A) Creatine Phosphate and (B) Sodium Pyrophosphate and polyphosphate (Graham´s Salt = Sodium Hexametaphosphate) in spiked samples. The bottom spectra in both A & B show the unspiked samples a few days after sample preparation. Note that some of the original peaks, shown in fig.SF1 are missing in the unspiked samples. These represented highly reactive compounds, such as ATP, Polyphosphate and Creatine Phosphate that depleted over time. Pyrophophate was still visible in both cases.

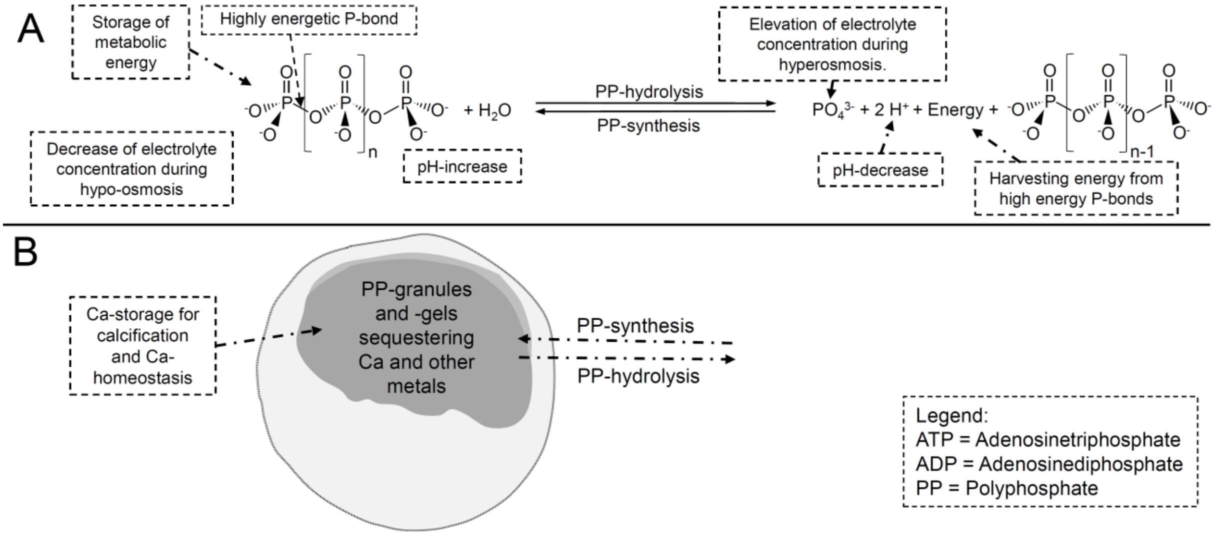

Fig. SF3: Summary of different possible metabolic functions of polyphosphates in foraminifera. A: Different biochemical processes and metabolic functions through hydrolysis and synthesis of polyphosphates (PP). "n" indicates is the length of the polyphosphate chain. B: Schematic representation of an acidocalcisome, filled with PP.

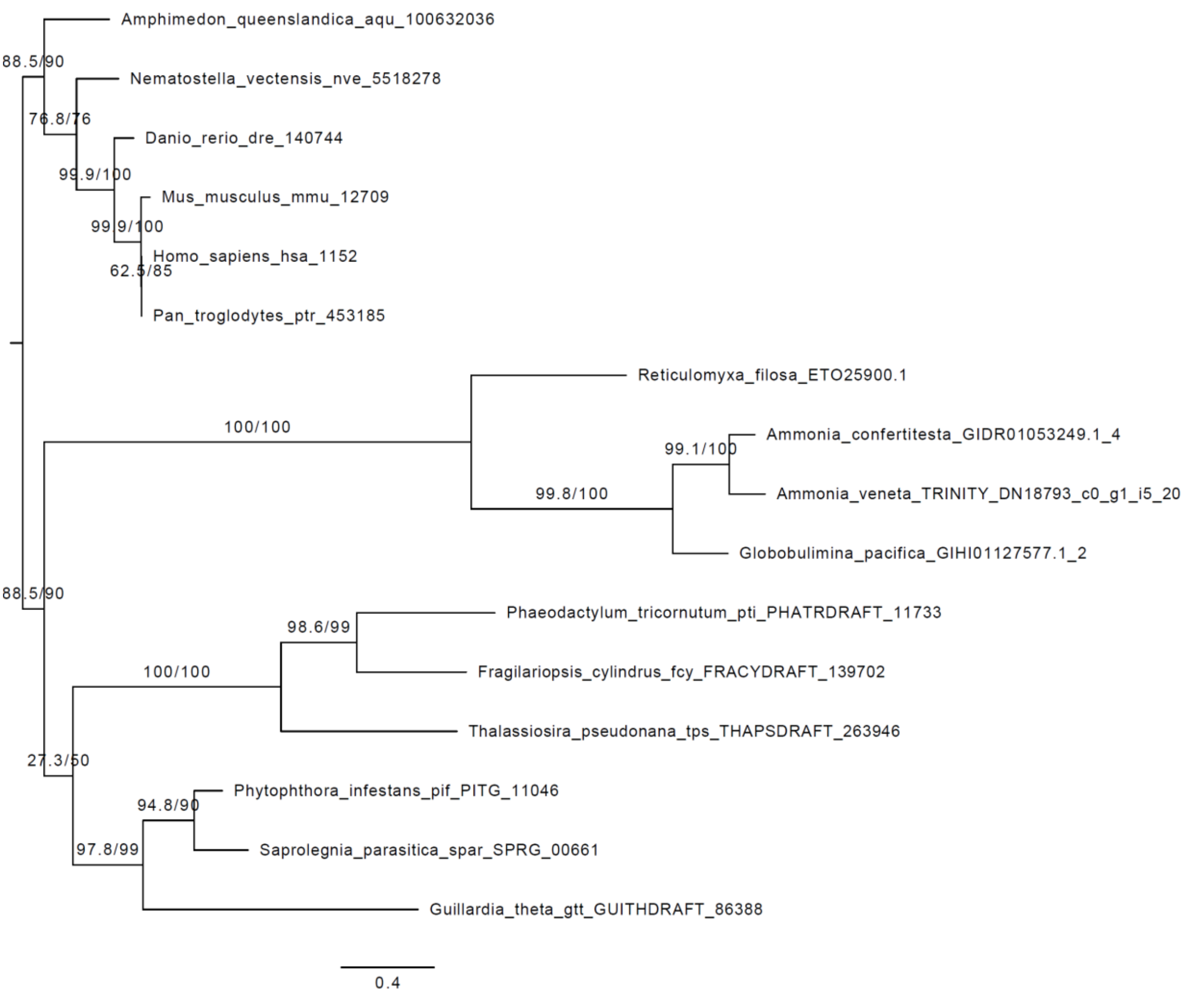

Fig.SF4: Phylogenetic tree of creatine kinase homologs in foraminifera. The phylogeny is based on representative protein sequences. Species names are shown followed by the sequence IDs (and a number of the peptide). The homolog of *Ammonia venata* derived from an assembly produced in the current study. Numbers at the branches represent ultrafast bootstrap and SH-aLRT scores, respectively. The tree was rooted between the taxonomic groups Sar (+ Guillardia theta) and Opisthokonta.

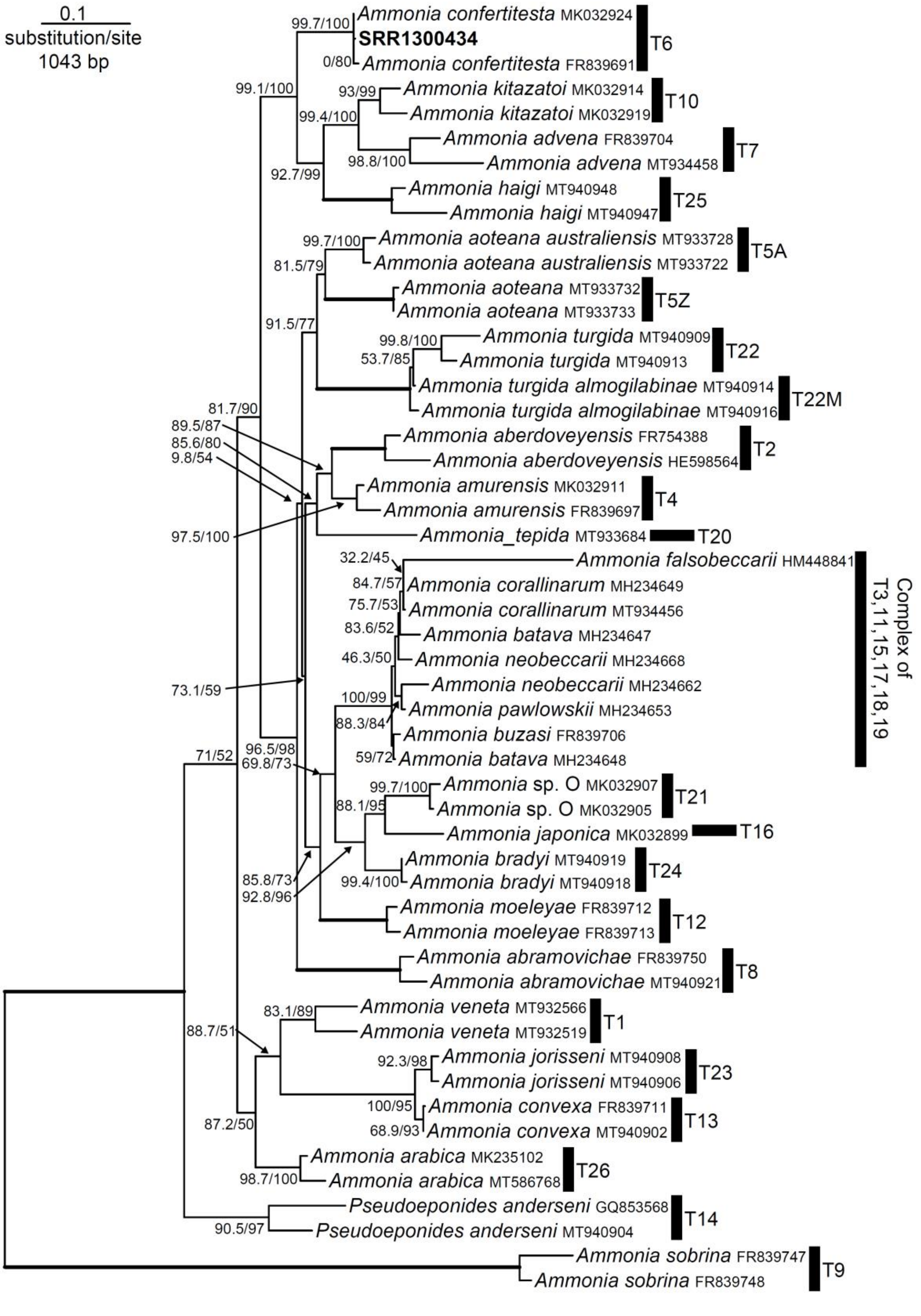

Fig.SF5: Phylogeny of partial 18S rRNA (1,043 bp) in genus *Ammonia*. Phylotype of *Ammonia*[9] are shown on right side. Numbers on the nodes show bootstrap values obtained from ultrafast bootstrap approximation[13] and SH-aLRT[14], respectively. Bold line indicate 100/100. 18s rRNA identified from published transcriptome data of *Ammonia* sp. (SRR1300434) is shown in bold character.

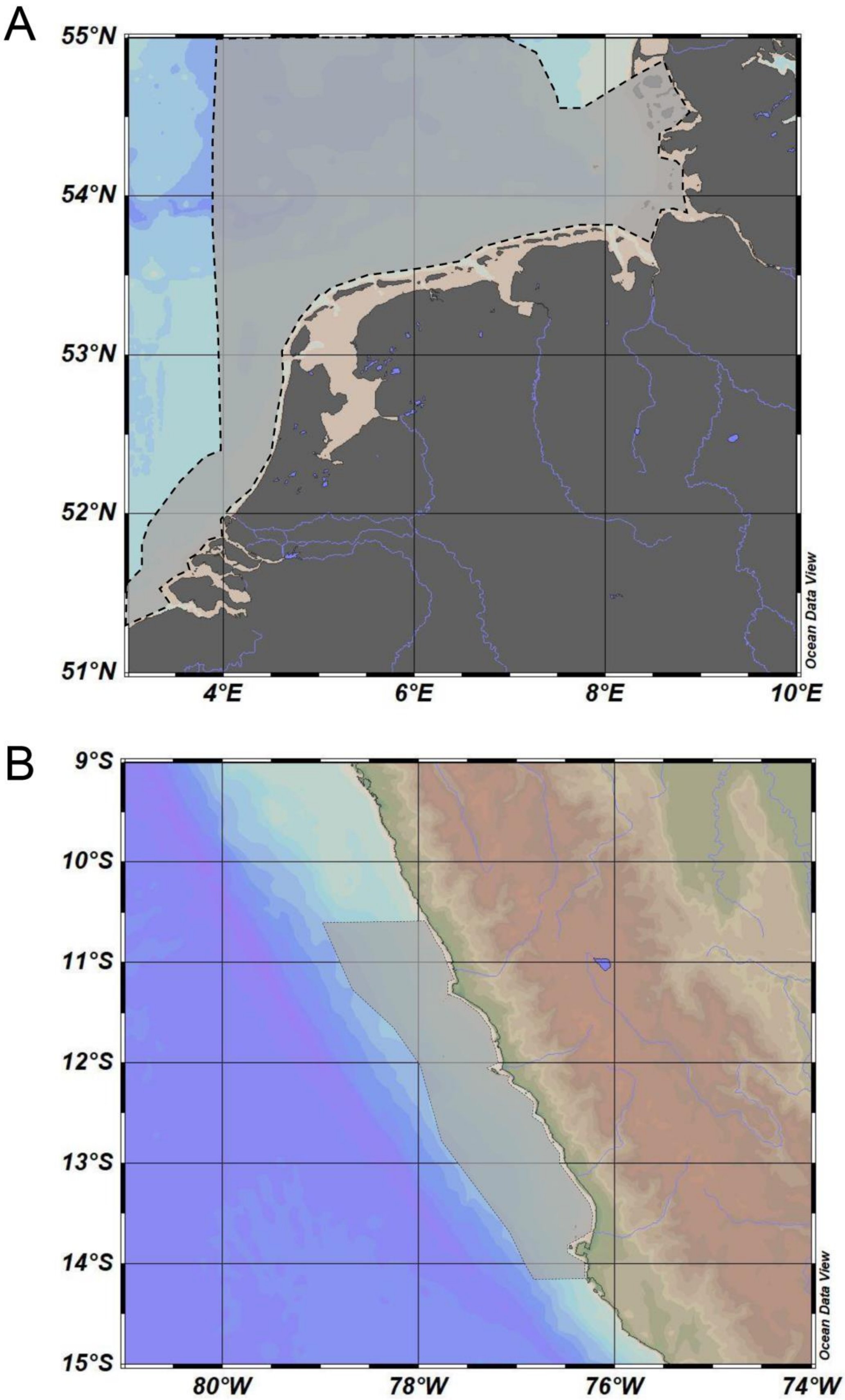

Fig.SF6: Regions of interest for calculating budgets of foraminiferal phosphate storage at (A) the Southern North Sea and (B) the Peruvian margin. Maps have been created, using ocean data view[18].

Tab.ST1: Bottom water O_2 concentrations and O_2 penetration depths into the pore water. At some locations, not literature data was available and we took data from similar environments. *: Data has been taken from the Janssand intertidal area. **: O_2 pore water profiles have been taken in situ but in deeper water depths than the analysed foraminifera (> 800 m). Bottom water O_2 concentrations were higher at the deeper stations and we expect the O_2 penetration depths to be even lower at the relevant shallower stations. ***: The O_2 penetration depths have been taken from another region at the Mid-Atlantic-Ridge in similar water depths with similar environmental conditions.

Location	Type	Bottom water O_2 concentrations (µmol/kg)	O_2 penetration depths (mm)
Sagami Bay (Japan)	Hypoxic	55 - 59[19]	2.6 – 17.8 (ø = 6.6 ± 2.5)[19]
Friedrichskoog (intertidal mudflat)	Well ventilated bottom water with low O_2 penetration	Saturated[20,*]	Low tide: 3 - 10[20,*]<br>High tide: 8 - 55[20,*]
Bedford Basin	Seasonally hypoxic to anoxic	2 - 290 (depending on season)[21]	~1[21]
Peruvian OMZ	Permanently suboxic to anoxic	1 - 13 (depending on water depth between 100 - 700 m)[22]	< 4[23,**]
Rainbow Vent Field (Mid-Atlantic-Ridge)	Well ventilated	242 - 246 between 2100 and 3100 m water depth[24]	Up to 8000 (i.e. 8 m)[25,***]

Tab.2 (Methods only). Sampling Sites

Station	Lattitude (°N)	Longitude (°W)	Water depth (m)	Region
HB1	35.3202	220.3658	0	Hirakata Bay (Japan)
NSB 2019 (for P content)	35.0017	220.8634	1405	Sagami Bay (Japan)
NSB 2022 & 2023 (for Cryo-SEM)	35.0037	220.8648	1398	Sagami Bay (Japan)
M176/2 St.6	36.2503	33.8598	2290	Rainbow Vent field (MAR)
M176/2 St.10	36.2783	33.8549	2670	Rainbow Vent field (MAR)
M176/2 St.13	36.2583	33.8365	2545	Rainbow Vent field (MAR)
M176/2 St.14	36.2356	33.8347	2670	Rainbow Vent field (MAR)
M176/2 St.16	36.2196	33.7851	3061	Rainbow Vent field (MAR)
M176/2 St.24	36.5563	33.5774	2930	Rainbow Vent field (MAR)
BB March 2022 MC1	44.8144	63.8056	73	Bedford Basin (Canada)
BB March 2022 MC2	44.9353	63.8056	49	Bedford Basin (Kanada)
BB March 2022 MC3	44.9206	63.8958	34	Bedford Basin (Kanada)
FK1	54.0302	8.8362	0	Friedrichskoog (Germany)

Tab.3 (Methods only): Best resembling shapes for the biovolume determination of foraminiferal species used in this study.

Species	Shape
Hoeglundina elegans	Prolate spheroid
Rhizammina algaeformis (fragment)	Cylinder
Cibicidoides wuellerstorfi	Tri axial ellipsoid
Cibicides mundulus	Prolate spheroid
Bolivina spissa	Cone with elliptic base
Chilostomella ovoidea	Prolate spheroid
Ammonia confertitesta	Half prolate spheroid
Ammonia veneta	Half prolate spheroid
Globobulimina affinis	Prolate spheroid
Labrospira crassimarga	Half prolate spheroid
Spiroplectammina biformis	Cone
Eggerella advena	Cone
Stainforthia fusiformis	Cone
Haynesina germanica	Half prolate spheroid
Uvigerina akitaensis	Prolate spheroid
Elphidium bartletti	Half prolate spheroid

Tab.ST4: Mean cell volumes and intracellular phosphate content in benthic foraminifera. Intracellular phosphate-contents are reported as individual specific (total content per individual, pmol ind^{-1}) and volume specific (intracellular content, mmol L^{-1}). ‘ # Ind.’ refers to the number of specimens used for each analysis. Samples marked with an asterisk (*) included only one specimen, so it was not possible to calculate an error for the mean cell volume. SE is the standard error of the mean.

Species	# Ind.	Mean cell volume (μm^3)	1se	Individual phosphate content (pmol ind^{-1})	Intracellular phosphate concentration (mmol L^{-1})	Region	Station
Ammonia confertitesta	40	9.25E+06	6.91E+05	525	56.8	Friedrichskoog	FK1
Ammonia confertitesta	36	1.06E+07	7.25E+05	557	52.3	Friedrichskoog	FK1
Ammonia confertitesta	39	8.07E+06	6.54E+05	153	18.9	Friedrichskoog	FK1
Ammonia veneta (oxic)	70	3.63E+06	2.27E+06	20	2.6	Cultures	Oxic
Ammonia veneta (anoxic)	75	3.72E+06	1.26E+06	10	5.4	Cultures	Anoxic
Bolivina spissa	30	3.49E+06	2.35E+05	83	23.8	Sagami Bay	NSB 2019
Chilostomella ovoidea	28	8.06E+06	5.84E+05	1	0.1	Sagami Bay	NSB 2019
Cibicides mundulus	9	6.09E+07	1.12E+07	3	0.0	Rainbow Vent field (MAR)	M176/2 St.06
Cibicides mundulus	13	7.94E+07	9.02E+06	2	0.0	Rainbow Vent field (MAR)	M176/2 St.10
Cibicides mundulus	10	5.95E+07	9.56E+06	4	0.1	Rainbow Vent field (MAR)	M176/2 St.13
Cibicides mundulus	12	7.20E+07	9.43E+06	1	0.0	Rainbow Vent field (MAR)	M176/2 St.13
Cibicidoides wuellerstorfi	11	2.64E+07	2.80E+06	5	0.2	Rainbow Vent field (MAR)	M176/2 St.06
Cibicidoides wuellerstorfi	6	1.81E+07	6.90E+06	4	0.2	Rainbow Vent field (MAR)	M176/2 St.06
Cibicidoides wuellerstorfi	11	1.48E+07	1.94E+06	4	0.3	Rainbow Vent field (MAR)	M176/2 St.10
Cibicidoides wuellerstorfi	11	1.72E+07	1.98E+06	0	0.0	Rainbow Vent field (MAR)	mixed stations
Eggerella advena	15	2.59E+06	1.56E+05	31	12.1	Bedford Basin	BB March 2022 MC3
Elphidium bartletti	8	3.25E+07	7.09E+06	497	15.3	Bedford Basin	BB March 2022 MC3
Elphidium bartletti	11	2.63E+07	3.45E+06	459	17.4	Bedford Basin	BB March 2022 MC3
Globobulimina affinis	7	9.62E+07	8.94E+06	600	6.2	Bedford Basin	BB March 2022 MC2
Globobulimina affnis	10	1.93E+08	3.05E+07	936	4.8	Sagami Bay	NSB 2019
Globobulimina affnis	12	1.58E+08	2.81E+07	1220	7.7	Sagami Bay	NSB 2019
Haynesina germanica	39	1.23E+07	6.24E+05	181	14.7	Friedrichskoog	FK1
Haynesina germanica	36	1.18E+07	7.40E+05	142	12.0	Friedrichskoog	FK1

Haynesina germanica	42	1.17E+07	5.97E+05	146	12.5	Friedrichskoog	FK1
Haynesina germanica	41	1.01E+07	5.90E+05	190	18.8	Friedrichskoog	FK1
Hoeglundina elegans	6	1.28E+08	4.60E+07	88	0.7	Rainbow Vent field (MAR)	M176/2 St.24
Labrospira crassimarga	3	3.97E+07	1.82E+07	56	1.4	Bedford Basin	BB March 2022 MC3
Rhizammina algaefirmis (fragment)	1	7.40E+08	*	108	0.1	Rainbow Vent field (MAR)	M176/2 St.16
Spiroplectammina biformis	35	2.14E+06	1.17E+05	20	9.2	Bedford Basin	BB March 2022 MC3
Spiroplectammina biformis	24	1.90E+06	1.24E+05	18	9.5	Bedford Basin	BB March 2022 MC3
Stainforthia fusiformis	9	6.26E+05	7.16E+04	14	22.4	Bedford Basin	BB March 2022 MC1
Stainforthia fusiformis	56	8.34E+05	4.00E+04	6	6.9	Bedford Basin	BB March 2022 MC2
Stainforthia fusiformis	70	7.97E+05	3.08E+04	6	7.6	Bedford Basin	BB March 2022 MC2
Uvigerina akitaensis	12	1.59E+07	1.83E+06	206	13.0	Sagami Bay	NSB 2019
Uvigerina akitaensis	12	1.41E+07	2.66E+06	200	14.2	Sagami Bay	NSB 2019

Tab.ST5: Biovolumes of individual benthic foraminifera determined for this study. For additional information about the sampling station see Tab. 1.

Species	Cell volume (μm^3)	Region	Station
Ammonia confertitesta	8.37E+06	Friedrichskoog	FK1
Ammonia confertitesta	6.60E+06	Friedrichskoog	FK1
Ammonia confertitesta	1.07E+07	Friedrichskoog	FK1
Ammonia confertitesta	8.33E+06	Friedrichskoog	FK1
Ammonia confertitesta	9.19E+06	Friedrichskoog	FK1
Ammonia confertitesta	9.07E+06	Friedrichskoog	FK1
Ammonia confertitesta	6.42E+06	Friedrichskoog	FK1
Ammonia confertitesta	9.70E+06	Friedrichskoog	FK1
Ammonia confertitesta	1.27E+07	Friedrichskoog	FK1
Ammonia confertitesta	4.73E+06	Friedrichskoog	FK1
Ammonia confertitesta	7.22E+06	Friedrichskoog	FK1
Ammonia confertitesta	8.85E+06	Friedrichskoog	FK1
Ammonia confertitesta	9.30E+06	Friedrichskoog	FK1
Ammonia confertitesta	8.19E+06	Friedrichskoog	FK1
Ammonia confertitesta	7.29E+06	Friedrichskoog	FK1
Ammonia confertitesta	1.05E+07	Friedrichskoog	FK1
Ammonia confertitesta	8.83E+06	Friedrichskoog	FK1
Ammonia confertitesta	5.30E+06	Friedrichskoog	FK1
Ammonia confertitesta	7.49E+06	Friedrichskoog	FK1
Ammonia confertitesta	6.13E+06	Friedrichskoog	FK1
Ammonia confertitesta	9.35E+06	Friedrichskoog	FK1
Ammonia confertitesta	8.70E+06	Friedrichskoog	FK1
Ammonia confertitesta	7.84E+06	Friedrichskoog	FK1
Ammonia confertitesta	6.82E+06	Friedrichskoog	FK1
Ammonia confertitesta	5.61E+06	Friedrichskoog	FK1
Ammonia confertitesta	8.83E+06	Friedrichskoog	FK1
Ammonia confertitesta	7.95E+06	Friedrichskoog	FK1
Ammonia confertitesta	3.30E+07	Friedrichskoog	FK1
Ammonia confertitesta	6.88E+06	Friedrichskoog	FK1
Ammonia confertitesta	6.56E+06	Friedrichskoog	FK1
Ammonia confertitesta	1.07E+07	Friedrichskoog	FK1
Ammonia confertitesta	9.88E+06	Friedrichskoog	FK1
Ammonia confertitesta	1.05E+07	Friedrichskoog	FK1
Ammonia confertitesta	1.08E+07	Friedrichskoog	FK1
Ammonia confertitesta	7.38E+06	Friedrichskoog	FK1
Ammonia confertitesta	1.55E+07	Friedrichskoog	FK1
Ammonia confertitesta	9.40E+06	Friedrichskoog	FK1
Ammonia confertitesta	1.03E+07	Friedrichskoog	FK1
Ammonia confertitesta	1.10E+07	Friedrichskoog	FK1
Ammonia confertitesta	8.24E+06	Friedrichskoog	FK1
Ammonia confertitesta	2.40E+07	Friedrichskoog	FK1
Ammonia confertitesta	1.08E+07	Friedrichskoog	FK1
Ammonia confertitesta	1.24E+07	Friedrichskoog	FK1
Ammonia confertitesta	1.39E+07	Friedrichskoog	FK1
Ammonia confertitesta	1.02E+07	Friedrichskoog	FK1

Ammonia confertitesta	1.39E+07	Friedrichskoog	FK1
Ammonia confertitesta	1.57E+07	Friedrichskoog	FK1
Ammonia confertitesta	7.58E+06	Friedrichskoog	FK1
Ammonia confertitesta	3.29E+06	Friedrichskoog	FK1
Ammonia confertitesta	4.71E+06	Friedrichskoog	FK1
Ammonia confertitesta	1.17E+07	Friedrichskoog	FK1
Ammonia confertitesta	1.21E+07	Friedrichskoog	FK1
Ammonia confertitesta	8.16E+06	Friedrichskoog	FK1
Ammonia confertitesta	6.99E+06	Friedrichskoog	FK1
Ammonia confertitesta	1.34E+07	Friedrichskoog	FK1
Ammonia confertitesta	1.17E+07	Friedrichskoog	FK1
Ammonia confertitesta	6.53E+06	Friedrichskoog	FK1
Ammonia confertitesta	7.40E+06	Friedrichskoog	FK1
Ammonia confertitesta	5.13E+06	Friedrichskoog	FK1
Ammonia confertitesta	8.15E+06	Friedrichskoog	FK1
Ammonia confertitesta	1.32E+07	Friedrichskoog	FK1
Ammonia confertitesta	7.36E+06	Friedrichskoog	FK1
Ammonia confertitesta	1.03E+07	Friedrichskoog	FK1
Ammonia confertitesta	1.77E+07	Friedrichskoog	FK1
Ammonia confertitesta	8.60E+06	Friedrichskoog	FK1
Ammonia confertitesta	6.41E+06	Friedrichskoog	FK1
Ammonia confertitesta	7.75E+06	Friedrichskoog	FK1
Ammonia confertitesta	6.11E+06	Friedrichskoog	FK1
Ammonia confertitesta	6.07E+06	Friedrichskoog	FK1
Ammonia confertitesta	1.17E+07	Friedrichskoog	FK1
Ammonia confertitesta	8.95E+06	Friedrichskoog	FK1
Ammonia confertitesta	1.61E+07	Friedrichskoog	FK1
Ammonia confertitesta	9.39E+06	Friedrichskoog	FK1
Ammonia confertitesta	1.47E+07	Friedrichskoog	FK1
Ammonia confertitesta	1.59E+07	Friedrichskoog	FK1
Ammonia confertitesta	1.52E+07	Friedrichskoog	FK1
Ammonia confertitesta	4.15E+06	Friedrichskoog	FK1
Ammonia confertitesta	5.17E+06	Friedrichskoog	FK1
Ammonia confertitesta	3.69E+06	Friedrichskoog	FK1
Ammonia confertitesta	4.77E+06	Friedrichskoog	FK1
Ammonia confertitesta	8.71E+06	Friedrichskoog	FK1
Ammonia confertitesta	6.81E+06	Friedrichskoog	FK1
Ammonia confertitesta	6.84E+06	Friedrichskoog	FK1
Ammonia confertitesta	5.32E+06	Friedrichskoog	FK1
Ammonia confertitesta	8.97E+06	Friedrichskoog	FK1
Ammonia confertitesta	9.33E+06	Friedrichskoog	FK1
Ammonia confertitesta	6.91E+06	Friedrichskoog	FK1
Ammonia confertitesta	7.03E+06	Friedrichskoog	FK1
Ammonia confertitesta	3.30E+06	Friedrichskoog	FK1
Ammonia confertitesta	5.95E+06	Friedrichskoog	FK1
Ammonia confertitesta	2.75E+07	Friedrichskoog	FK1
Ammonia confertitesta	1.17E+07	Friedrichskoog	FK1
Ammonia confertitesta	8.32E+06	Friedrichskoog	FK1
Ammonia confertitesta	7.19E+06	Friedrichskoog	FK1

Ammonia confertitesta	8.95E+06	Friedrichskoog	FK1
Ammonia confertitesta	7.12E+06	Friedrichskoog	FK1
Ammonia confertitesta	7.31E+06	Friedrichskoog	FK1
Ammonia confertitesta	7.31E+06	Friedrichskoog	FK1
Ammonia confertitesta	5.43E+06	Friedrichskoog	FK1
Ammonia confertitesta	1.02E+07	Friedrichskoog	FK1
Ammonia confertitesta	4.24E+06	Friedrichskoog	FK1
Ammonia confertitesta	8.31E+06	Friedrichskoog	FK1
Ammonia confertitesta	9.77E+06	Friedrichskoog	FK1
Ammonia confertitesta	8.65E+06	Friedrichskoog	FK1
Ammonia confertitesta	1.20E+07	Friedrichskoog	FK1
Ammonia confertitesta	1.15E+07	Friedrichskoog	FK1
Ammonia confertitesta	8.20E+06	Friedrichskoog	FK1
Ammonia confertitesta	1.22E+07	Friedrichskoog	FK1
Ammonia confertitesta	8.84E+06	Friedrichskoog	FK1
Ammonia confertitesta	5.89E+06	Friedrichskoog	FK1
Ammonia confertitesta	5.44E+06	Friedrichskoog	FK1
Ammonia confertitesta	1.40E+07	Friedrichskoog	FK1
Ammonia confertitesta	5.93E+06	Friedrichskoog	FK1
Ammonia confertitesta	7.44E+06	Friedrichskoog	FK1
Ammonia confertitesta	4.29E+06	Friedrichskoog	FK1
Ammonia veneta	2.33E+06	Cultures	Anoxic
Ammonia veneta	3.49E+06	Cultures	Anoxic
Ammonia veneta	2.63E+06	Cultures	Anoxic
Ammonia veneta	1.73E+06	Cultures	Anoxic
Ammonia veneta	1.49E+06	Cultures	Anoxic
Ammonia veneta	4.61E+06	Cultures	Anoxic
Ammonia veneta	3.46E+06	Cultures	Anoxic
Ammonia veneta	4.61E+06	Cultures	Anoxic
Ammonia veneta	5.96E+06	Cultures	Anoxic
Ammonia veneta	5.62E+06	Cultures	Anoxic
Ammonia veneta	4.45E+06	Cultures	Anoxic
Ammonia veneta	6.65E+06	Cultures	Anoxic
Ammonia veneta	4.40E+06	Cultures	Anoxic
Ammonia veneta	7.07E+06	Cultures	Anoxic
Ammonia veneta	4.19E+06	Cultures	Anoxic
Ammonia veneta	4.32E+06	Cultures	Anoxic
Ammonia veneta	3.64E+06	Cultures	Anoxic
Ammonia veneta	4.11E+06	Cultures	Anoxic
Ammonia veneta	4.23E+06	Cultures	Anoxic
Ammonia veneta	4.68E+06	Cultures	Anoxic
Ammonia veneta	5.50E+06	Cultures	Anoxic
Ammonia veneta	3.38E+06	Cultures	Anoxic
Ammonia veneta	4.68E+06	Cultures	Anoxic
Ammonia veneta	3.45E+06	Cultures	Anoxic
Ammonia veneta	4.63E+06	Cultures	Anoxic
Ammonia veneta	3.71E+06	Cultures	Anoxic
Ammonia veneta	2.98E+06	Cultures	Anoxic
Ammonia veneta	3.11E+06	Cultures	Anoxic

Ammonia veneta	1.52E+06	Cultures	Anoxic
Ammonia veneta	3.04E+06	Cultures	Anoxic
Ammonia veneta	3.62E+06	Cultures	Anoxic
Ammonia veneta	3.64E+06	Cultures	Anoxic
Ammonia veneta	3.62E+06	Cultures	Anoxic
Ammonia veneta	2.96E+06	Cultures	Anoxic
Ammonia veneta	3.70E+06	Cultures	Anoxic
Ammonia veneta	4.26E+06	Cultures	Anoxic
Ammonia veneta	3.52E+06	Cultures	Anoxic
Ammonia veneta	2.67E+06	Cultures	Anoxic
Ammonia veneta	3.17E+06	Cultures	Anoxic
Ammonia veneta	4.18E+06	Cultures	Anoxic
Ammonia veneta	3.08E+06	Cultures	Anoxic
Ammonia veneta	3.59E+06	Cultures	Anoxic
Ammonia veneta	2.24E+06	Cultures	Anoxic
Ammonia veneta	3.11E+06	Cultures	Anoxic
Ammonia veneta	2.94E+06	Cultures	Anoxic
Ammonia veneta	8.47E+06	Cultures	Anoxic
Ammonia veneta	4.44E+06	Cultures	Anoxic
Ammonia veneta	6.26E+06	Cultures	Anoxic
Ammonia veneta	4.30E+06	Cultures	Anoxic
Ammonia veneta	4.10E+06	Cultures	Anoxic
Ammonia veneta	3.43E+06	Cultures	Anoxic
Ammonia veneta	3.33E+06	Cultures	Anoxic
Ammonia veneta	3.72E+06	Cultures	Anoxic
Ammonia veneta	3.51E+06	Cultures	Anoxic
Ammonia veneta	4.41E+06	Cultures	Anoxic
Ammonia veneta	3.26E+06	Cultures	Anoxic
Ammonia veneta	3.19E+06	Cultures	Anoxic
Ammonia veneta	3.10E+06	Cultures	Anoxic
Ammonia veneta	1.98E+06	Cultures	Anoxic
Ammonia veneta	1.86E+06	Cultures	Anoxic
Ammonia veneta	3.16E+06	Cultures	Anoxic
Ammonia veneta	4.43E+06	Cultures	Anoxic
Ammonia veneta	4.66E+06	Cultures	Anoxic
Ammonia veneta	2.74E+06	Cultures	Anoxic
Ammonia veneta	1.88E+06	Cultures	Anoxic
Ammonia veneta	4.69E+06	Cultures	Anoxic
Ammonia veneta	3.13E+06	Cultures	Anoxic
Ammonia veneta	2.26E+06	Cultures	Anoxic
Ammonia veneta	3.29E+06	Cultures	Anoxic
Ammonia veneta	3.29E+06	Cultures	Anoxic
Ammonia veneta	4.40E+06	Cultures	Anoxic
Ammonia veneta	3.16E+06	Cultures	Anoxic
Ammonia veneta	4.13E+06	Cultures	Anoxic
Ammonia veneta	1.97E+06	Cultures	Anoxic
Ammonia veneta	2.18E+06	Cultures	Anoxic
Ammonia veneta	5.92E+06	Cultures	Oxic
Ammonia veneta	3.08E+06	Cultures	Oxic

Ammonia veneta	7.57E+06	Cultures	Oxic
Ammonia veneta	4.45E+06	Cultures	Oxic
Ammonia veneta	2.43E+06	Cultures	Oxic
Ammonia veneta	4.52E+06	Cultures	Oxic
Ammonia veneta	2.02E+06	Cultures	Oxic
Ammonia veneta	6.80E+06	Cultures	Oxic
Ammonia veneta	2.96E+06	Cultures	Oxic
Ammonia veneta	3.04E+06	Cultures	Oxic
Ammonia veneta	2.65E+06	Cultures	Oxic
Ammonia veneta	2.28E+06	Cultures	Oxic
Ammonia veneta	3.01E+06	Cultures	Oxic
Ammonia veneta	2.96E+06	Cultures	Oxic
Ammonia veneta	2.07E+06	Cultures	Oxic
Ammonia veneta	2.40E+06	Cultures	Oxic
Ammonia veneta	3.10E+06	Cultures	Oxic
Ammonia veneta	4.73E+06	Cultures	Oxic
Ammonia veneta	4.90E+06	Cultures	Oxic
Ammonia veneta	3.64E+06	Cultures	Oxic
Ammonia veneta	4.26E+06	Cultures	Oxic
Ammonia veneta	4.82E+06	Cultures	Oxic
Ammonia veneta	2.90E+06	Cultures	Oxic
Ammonia veneta	3.90E+06	Cultures	Oxic
Ammonia veneta	2.24E+06	Cultures	Oxic
Ammonia veneta	1.73E+06	Cultures	Oxic
Ammonia veneta	2.45E+06	Cultures	Oxic
Ammonia veneta	1.02E+07	Cultures	Oxic
Ammonia veneta	3.91E+06	Cultures	Oxic
Ammonia veneta	6.03E+06	Cultures	Oxic
Ammonia veneta	1.06E+07	Cultures	Oxic
Ammonia veneta	1.58E+07	Cultures	Oxic
Ammonia veneta	2.02E+06	Cultures	Oxic
Ammonia veneta	3.18E+06	Cultures	Oxic
Ammonia veneta	1.67E+06	Cultures	Oxic
Ammonia veneta	3.36E+06	Cultures	Oxic
Ammonia veneta	1.60E+06	Cultures	Oxic
Ammonia veneta	2.15E+06	Cultures	Oxic
Ammonia veneta	1.90E+06	Cultures	Oxic
Ammonia veneta	3.05E+06	Cultures	Oxic
Ammonia veneta	2.40E+06	Cultures	Oxic
Ammonia veneta	2.47E+06	Cultures	Oxic
Ammonia veneta	1.08E+06	Cultures	Oxic
Ammonia veneta	1.51E+06	Cultures	Oxic
Ammonia veneta	4.08E+06	Cultures	Oxic
Ammonia veneta	2.07E+06	Cultures	Oxic
Ammonia veneta	3.59E+06	Cultures	Oxic
Ammonia veneta	4.50E+06	Cultures	Oxic
Ammonia veneta	2.87E+06	Cultures	Oxic
Ammonia veneta	3.61E+06	Cultures	Oxic
Ammonia veneta	2.80E+06	Cultures	Oxic

Ammonia veneta	3.35E+06	Cultures	Oxic
Ammonia veneta	3.20E+06	Cultures	Oxic
Ammonia veneta	5.35E+06	Cultures	Oxic
Ammonia veneta	3.74E+06	Cultures	Oxic
Ammonia veneta	3.37E+06	Cultures	Oxic
Ammonia veneta	3.16E+06	Cultures	Oxic
Ammonia veneta	4.93E+06	Cultures	Oxic
Ammonia veneta	3.31E+06	Cultures	Oxic
Ammonia veneta	2.48E+06	Cultures	Oxic
Ammonia veneta	3.94E+06	Cultures	Oxic
Ammonia veneta	2.37E+06	Cultures	Oxic
Ammonia veneta	3.05E+06	Cultures	Oxic
Ammonia veneta	2.01E+06	Cultures	Oxic
Ammonia veneta	1.99E+06	Cultures	Oxic
Ammonia veneta	2.79E+06	Cultures	Oxic
Ammonia veneta	2.65E+06	Cultures	Oxic
Ammonia veneta	3.02E+06	Cultures	Oxic
Ammonia veneta	3.26E+06	Cultures	Oxic
Ammonia veneta	2.82E+06	Cultures	Oxic
Bolivina spissa	2.82E+06	Sagami Bay	NSB 2019
Bolivina spissa	2.55E+06	Sagami Bay	NSB 2019
Bolivina spissa	3.24E+06	Sagami Bay	NSB 2019
Bolivina spissa	4.58E+06	Sagami Bay	NSB 2019
Bolivina spissa	3.78E+06	Sagami Bay	NSB 2019
Bolivina spissa	2.60E+06	Sagami Bay	NSB 2019
Bolivina spissa	4.51E+06	Sagami Bay	NSB 2019
Bolivina spissa	2.91E+06	Sagami Bay	NSB 2019
Bolivina spissa	3.78E+06	Sagami Bay	NSB 2019
Bolivina spissa	2.68E+06	Sagami Bay	NSB 2019
Bolivina spissa	2.92E+06	Sagami Bay	NSB 2019
Bolivina spissa	3.83E+06	Sagami Bay	NSB 2019
Bolivina spissa	2.93E+06	Sagami Bay	NSB 2019
Bolivina spissa	2.36E+06	Sagami Bay	NSB 2019
Bolivina spissa	3.34E+06	Sagami Bay	NSB 2019
Bolivina spissa	4.65E+06	Sagami Bay	NSB 2019
Bolivina spissa	2.94E+06	Sagami Bay	NSB 2019
Bolivina spissa	2.44E+06	Sagami Bay	NSB 2019
Bolivina spissa	3.83E+06	Sagami Bay	NSB 2019
Bolivina spissa	7.34E+06	Sagami Bay	NSB 2019
Bolivina spissa	2.34E+06	Sagami Bay	NSB 2019
Bolivina spissa	4.16E+06	Sagami Bay	NSB 2019
Bolivina spissa	3.51E+06	Sagami Bay	NSB 2019
Bolivina spissa	3.58E+06	Sagami Bay	NSB 2019
Bolivina spissa	3.01E+06	Sagami Bay	NSB 2019
Bolivina spissa	1.88E+06	Sagami Bay	NSB 2019
Bolivina spissa	2.22E+06	Sagami Bay	NSB 2019
Bolivina spissa	4.03E+06	Sagami Bay	NSB 2019
Bolivina spissa	7.40E+06	Sagami Bay	NSB 2019
Bolivina spissa	2.57E+06	Sagami Bay	NSB 2019

Chilostimella ovoidea	1.07E+07	Sagami Bay	NSB 2019
Chilostimella ovoidea	9.17E+06	Sagami Bay	NSB 2019
Chilostimella ovoidea	7.72E+06	Sagami Bay	NSB 2019
Chilostimella ovoidea	1.07E+07	Sagami Bay	NSB 2019
Chilostimella ovoidea	1.03E+07	Sagami Bay	NSB 2019
Chilostimella ovoidea	7.44E+06	Sagami Bay	NSB 2019
Chilostimella ovoidea	5.02E+06	Sagami Bay	NSB 2019
Chilostimella ovoidea	6.59E+06	Sagami Bay	NSB 2019
Chilostimella ovoidea	4.95E+06	Sagami Bay	NSB 2019
Chilostimella ovoidea	7.12E+06	Sagami Bay	NSB 2019
Chilostimella ovoidea	8.30E+06	Sagami Bay	NSB 2019
Chilostimella ovoidea	7.58E+06	Sagami Bay	NSB 2019
Chilostimella ovoidea	1.13E+07	Sagami Bay	NSB 2019
Chilostimella ovoidea	2.90E+06	Sagami Bay	NSB 2019
Chilostimella ovoidea	1.11E+07	Sagami Bay	NSB 2019
Chilostimella ovoidea	8.91E+06	Sagami Bay	NSB 2019
Chilostimella ovoidea	6.00E+06	Sagami Bay	NSB 2019
Chilostimella ovoidea	3.21E+06	Sagami Bay	NSB 2019
Chilostimella ovoidea	1.24E+07	Sagami Bay	NSB 2019
Chilostimella ovoidea	1.10E+07	Sagami Bay	NSB 2019
Chilostimella ovoidea	7.30E+06	Sagami Bay	NSB 2019
Chilostimella ovoidea	1.04E+07	Sagami Bay	NSB 2019
Chilostimella ovoidea	1.48E+07	Sagami Bay	NSB 2019
Chilostimella ovoidea	5.87E+06	Sagami Bay	NSB 2019
Chilostimella ovoidea	1.09E+07	Sagami Bay	NSB 2019
Chilostimella ovoidea	7.29E+06	Sagami Bay	NSB 2019
Chilostimella ovoidea	4.00E+06	Sagami Bay	NSB 2019
Chilostimella ovoidea	2.56E+06	Sagami Bay	NSB 2019
Cibicides mundulus	1.11E+08	Rainbow Vent field (MAR)	M176/2 St.06
Cibicides mundulus	1.18E+08	Rainbow Vent field (MAR)	M176/2 St.06
Cibicides mundulus	5.61E+07	Rainbow Vent field (MAR)	M176/2 St.06
Cibicides mundulus	3.43E+07	Rainbow Vent field (MAR)	M176/2 St.06
Cibicides mundulus	4.53E+07	Rainbow Vent field (MAR)	M176/2 St.06
Cibicides mundulus	5.08E+07	Rainbow Vent field (MAR)	M176/2 St.06
Cibicides mundulus	6.32E+07	Rainbow Vent field (MAR)	M176/2 St.06
Cibicides mundulus	5.54E+07	Rainbow Vent field (MAR)	M176/2 St.06
Cibicides mundulus	1.41E+07	Rainbow Vent field (MAR)	M176/2 St.06
Cibicides mundulus	1.10E+08	Rainbow Vent field (MAR)	M176/2 St.10
Cibicides mundulus	1.26E+08	Rainbow Vent field (MAR)	M176/2 St.10
Cibicides mundulus	6.63E+07	Rainbow Vent field (MAR)	M176/2 St.10

Cibicides mundulus	6.64E+07	Rainbow Vent field (MAR)	M176/2 St.10
Cibicides mundulus	1.26E+08	Rainbow Vent field (MAR)	M176/2 St.10
Cibicides mundulus	3.93E+07	Rainbow Vent field (MAR)	M176/2 St.10
Cibicides mundulus	9.46E+07	Rainbow Vent field (MAR)	M176/2 St.10
Cibicides mundulus	1.05E+08	Rainbow Vent field (MAR)	M176/2 St.10
Cibicides mundulus	7.56E+07	Rainbow Vent field (MAR)	M176/2 St.10
Cibicides mundulus	5.62E+07	Rainbow Vent field (MAR)	M176/2 St.10
Cibicides mundulus	9.19E+07	Rainbow Vent field (MAR)	M176/2 St.10
Cibicides mundulus	4.86E+07	Rainbow Vent field (MAR)	M176/2 St.10
Cibicides mundulus	2.54E+07	Rainbow Vent field (MAR)	M176/2 St.10
Cibicides mundulus	9.08E+07	Rainbow Vent field (MAR)	M176/2 St.13
Cibicides mundulus	8.26E+07	Rainbow Vent field (MAR)	M176/2 St.13
Cibicides mundulus	6.39E+07	Rainbow Vent field (MAR)	M176/2 St.13
Cibicides mundulus	5.69E+07	Rainbow Vent field (MAR)	M176/2 St.13
Cibicides mundulus	6.54E+07	Rainbow Vent field (MAR)	M176/2 St.13
Cibicides mundulus	1.50E+07	Rainbow Vent field (MAR)	M176/2 St.13
Cibicides mundulus	1.10E+08	Rainbow Vent field (MAR)	M176/2 St.13
Cibicides mundulus	4.70E+07	Rainbow Vent field (MAR)	M176/2 St.13
Cibicides mundulus	1.84E+07	Rainbow Vent field (MAR)	M176/2 St.13
Cibicides mundulus	4.41E+07	Rainbow Vent field (MAR)	M176/2 St.13
Cibicides mundulus	1.38E+08	Rainbow Vent field (MAR)	M176/2 St.13
Cibicides mundulus	1.02E+08	Rainbow Vent field (MAR)	M176/2 St.13
Cibicides mundulus	5.71E+07	Rainbow Vent field (MAR)	M176/2 St.13
Cibicides mundulus	4.37E+07	Rainbow Vent field (MAR)	M176/2 St.13
Cibicides mundulus	9.53E+07	Rainbow Vent field (MAR)	M176/2 St.13
Cibicides mundulus	1.02E+08	Rainbow Vent field (MAR)	M176/2 St.13
Cibicides mundulus	7.63E+07	Rainbow Vent field (MAR)	M176/2 St.13
Cibicides mundulus	3.10E+07	Rainbow Vent field (MAR)	M176/2 St.13

Cibicides mundulus	8.21E+07	Rainbow Vent field (MAR)	M176/2 St.13
Cibicides mundulus	5.25E+07	Rainbow Vent field (MAR)	M176/2 St.13
Cibicides mundulus	3.47E+07	Rainbow Vent field (MAR)	M176/2 St.13
Cibicides mundulus	5.04E+07	Rainbow Vent field (MAR)	M176/2 St.13
Cibicidoides wuellerstorfi	2.47E+07	Rainbow Vent field (MAR)	M176/2 pooled from mixed stations
Cibicidoides wuellerstorfi	1.84E+07	Rainbow Vent field (MAR)	M176/2 pooled from mixed stations
Cibicidoides wuellerstorfi	1.42E+07	Rainbow Vent field (MAR)	M176/2 pooled from mixed stations
Cibicidoides wuellerstorfi	1.45E+07	Rainbow Vent field (MAR)	M176/2 pooled from mixed stations
Cibicidoides wuellerstorfi	1.42E+07	Rainbow Vent field (MAR)	M176/2 pooled from mixed stations
Cibicidoides wuellerstorfi	1.41E+07	Rainbow Vent field (MAR)	M176/2 pooled from mixed stations
Cibicidoides wuellerstorfi	1.86E+07	Rainbow Vent field (MAR)	M176/2 pooled from mixed stations
Cibicidoides wuellerstorfi	4.40E+06	Rainbow Vent field (MAR)	M176/2 pooled from mixed stations
Cibicidoides wuellerstorfi	2.54E+07	Rainbow Vent field (MAR)	M176/2 pooled from mixed stations
Cibicidoides wuellerstorfi	2.69E+07	Rainbow Vent field (MAR)	M176/2 pooled from mixed stations
Cibicidoides wuellerstorfi	1.38E+07	Rainbow Vent field (MAR)	M176/2 pooled from mixed stations
Cibicidoides wuellerstorfi	3.55E+07	Rainbow Vent field (MAR)	M176/2 St.06
Cibicidoides wuellerstorfi	1.77E+07	Rainbow Vent field (MAR)	M176/2 St.06
Cibicidoides wuellerstorfi	3.84E+07	Rainbow Vent field (MAR)	M176/2 St.06
Cibicidoides wuellerstorfi	1.24E+07	Rainbow Vent field (MAR)	M176/2 St.06
Cibicidoides wuellerstorfi	1.95E+07	Rainbow Vent field (MAR)	M176/2 St.06
Cibicidoides wuellerstorfi	2.29E+07	Rainbow Vent field (MAR)	M176/2 St.06
Cibicidoides wuellerstorfi	2.97E+07	Rainbow Vent field (MAR)	M176/2 St.06
Cibicidoides wuellerstorfi	3.61E+07	Rainbow Vent field (MAR)	M176/2 St.06
Cibicidoides wuellerstorfi	3.31E+07	Rainbow Vent field (MAR)	M176/2 St.06
Cibicidoides wuellerstorfi	1.47E+07	Rainbow Vent field (MAR)	M176/2 St.06
Cibicidoides wuellerstorfi	3.03E+07	Rainbow Vent field (MAR)	M176/2 St.06
Cibicidoides wuellerstorfi	2.40E+07	Rainbow Vent field (MAR)	M176/2 St.06
Cibicidoides wuellerstorfi	5.49E+06	Rainbow Vent field (MAR)	M176/2 St.06

Cibicidoides wuellerstorfi	4.98E+07	Rainbow Vent field (MAR)	M176/2 St.06
Cibicidoides wuellerstorfi	7.48E+06	Rainbow Vent field (MAR)	M176/2 St.06
Cibicidoides wuellerstorfi	1.29E+07	Rainbow Vent field (MAR)	M176/2 St.06
Cibicidoides wuellerstorfi	8.66E+06	Rainbow Vent field (MAR)	M176/2 St.06
Cibicidoides wuellerstorfi	2.06E+07	Rainbow Vent field (MAR)	M176/2 St.10
Cibicidoides wuellerstorfi	1.10E+07	Rainbow Vent field (MAR)	M176/2 St.10
Cibicidoides wuellerstorfi	1.52E+07	Rainbow Vent field (MAR)	M176/2 St.10
Cibicidoides wuellerstorfi	1.29E+07	Rainbow Vent field (MAR)	M176/2 St.10
Cibicidoides wuellerstorfi	2.21E+07	Rainbow Vent field (MAR)	M176/2 St.10
Cibicidoides wuellerstorfi	2.43E+07	Rainbow Vent field (MAR)	M176/2 St.10
Cibicidoides wuellerstorfi	1.82E+07	Rainbow Vent field (MAR)	M176/2 St.10
Cibicidoides wuellerstorfi	6.28E+06	Rainbow Vent field (MAR)	M176/2 St.10
Cibicidoides wuellerstorfi	1.81E+07	Rainbow Vent field (MAR)	M176/2 St.10
Cibicidoides wuellerstorfi	9.25E+06	Rainbow Vent field (MAR)	M176/2 St.10
Cibicidoides wuellerstorfi	5.08E+06	Rainbow Vent field (MAR)	M176/2 St.10
Eggerella advena	2.25E+06	Bedford Basin	BB March 2022 MC3
Eggerella advena	1.83E+06	Bedford Basin	BB March 2022 MC3
Eggerella advena	2.43E+06	Bedford Basin	BB March 2022 MC3
Eggerella advena	2.43E+06	Bedford Basin	BB March 2022 MC3
Eggerella advena	1.77E+06	Bedford Basin	BB March 2022 MC3
Eggerella advena	2.09E+06	Bedford Basin	BB March 2022 MC3
Eggerella advena	3.68E+06	Bedford Basin	BB March 2022 MC3
Eggerella advena	2.83E+06	Bedford Basin	BB March 2022 MC3
Eggerella advena	3.78E+06	Bedford Basin	BB March 2022 MC3
Eggerella advena	2.33E+06	Bedford Basin	BB March 2022 MC3
Eggerella advena	3.02E+06	Bedford Basin	BB March 2022 MC3
Eggerella advena	3.08E+06	Bedford Basin	BB March 2022 MC3
Eggerella advena	2.46E+06	Bedford Basin	BB March 2022 MC3
Eggerella advena	2.08E+06	Bedford Basin	BB March 2022 MC3
Eggerella advena	2.77E+06	Bedford Basin	BB March 2022 MC3
Elphidium bartletti	4.23E+07	Bedford Basin	BB March 2022 MC3
Elphidium bartletti	8.76E+06	Bedford Basin	BB March 2022 MC3
Elphidium bartletti	2.38E+07	Bedford Basin	BB March 2022 MC3
Elphidium bartletti	6.23E+06	Bedford Basin	BB March 2022 MC3
Elphidium bartletti	3.03E+07	Bedford Basin	BB March 2022 MC3
Elphidium bartletti	2.85E+07	Bedford Basin	BB March 2022 MC3
Elphidium bartletti	3.48E+07	Bedford Basin	BB March 2022 MC3
Elphidium bartletti	2.65E+07	Bedford Basin	BB March 2022 MC3

Elphidium bartletti	1.97E+07	Bedford Basin	BB March 2022 MC3
Elphidium bartletti	4.02E+07	Bedford Basin	BB March 2022 MC3
Elphidium bartletti	2.81E+07	Bedford Basin	BB March 2022 MC3
Elphidium bartletti	5.49E+07	Bedford Basin	BB March 2022 MC3
Elphidium bartletti	5.95E+07	Bedford Basin	BB March 2022 MC3
Elphidium bartletti	3.70E+07	Bedford Basin	BB March 2022 MC3
Elphidium bartletti	1.00E+07	Bedford Basin	BB March 2022 MC3
Elphidium bartletti	4.58E+07	Bedford Basin	BB March 2022 MC3
Elphidium bartletti	6.18E+06	Bedford Basin	BB March 2022 MC3
Elphidium bartletti	2.56E+07	Bedford Basin	BB March 2022 MC3
Elphidium bartletti	2.07E+07	Bedford Basin	BB March 2022 MC3
Globobulimina affinis	1.04E+08	Bedford Basin	BB March 2022 MC2
Globobulimina affinis	1.15E+08	Bedford Basin	BB March 2022 MC2
Globobulimina affinis	5.88E+07	Bedford Basin	BB March 2022 MC2
Globobulimina affinis	1.19E+08	Bedford Basin	BB March 2022 MC2
Globobulimina affinis	1.17E+08	Bedford Basin	BB March 2022 MC2
Globobulimina affinis	7.60E+07	Bedford Basin	BB March 2022 MC2
Globobulimina affinis	8.30E+07	Bedford Basin	BB March 2022 MC2
Globobulimina affinis	2.27E+08	Sagami Bay	NSB 2019
Globobulimina affinis	2.76E+08	Sagami Bay	NSB 2019
Globobulimina affinis	7.60E+07	Sagami Bay	NSB 2019
Globobulimina affinis	8.55E+07	Sagami Bay	NSB 2019
Globobulimina affinis	2.53E+08	Sagami Bay	NSB 2019
Globobulimina affinis	1.25E+08	Sagami Bay	NSB 2019
Globobulimina affinis	2.67E+08	Sagami Bay	NSB 2019
Globobulimina affinis	1.64E+08	Sagami Bay	NSB 2019
Globobulimina affinis	1.00E+08	Sagami Bay	NSB 2019
Globobulimina affinis	3.57E+08	Sagami Bay	NSB 2019
Globobulimina affinis	2.54E+08	Sagami Bay	NSB 2019
Globobulimina affinis	8.59E+07	Sagami Bay	NSB 2019
Globobulimina affinis	9.24E+07	Sagami Bay	NSB 2019
Globobulimina affinis	5.09E+07	Sagami Bay	NSB 2019
Globobulimina affinis	7.97E+07	Sagami Bay	NSB 2019
Globobulimina affinis	1.92E+08	Sagami Bay	NSB 2019
Globobulimina affinis	1.05E+08	Sagami Bay	NSB 2019
Globobulimina affinis	1.07E+08	Sagami Bay	NSB 2019
Globobulimina affinis	2.08E+08	Sagami Bay	NSB 2019
Globobulimina affinis	2.44E+08	Sagami Bay	NSB 2019
Globobulimina affinis	9.99E+07	Sagami Bay	NSB 2019
Globobulimina affinis	3.77E+08	Sagami Bay	NSB 2019
Haynesina germanica	7.81E+06	Friedrichskoog	FK1
Haynesina germanica	1.33E+07	Friedrichskoog	FK1
Haynesina germanica	9.15E+06	Friedrichskoog	FK1
Haynesina germanica	1.32E+07	Friedrichskoog	FK1
Haynesina germanica	6.81E+06	Friedrichskoog	FK1
Haynesina germanica	1.09E+07	Friedrichskoog	FK1
Haynesina germanica	1.08E+07	Friedrichskoog	FK1
Haynesina germanica	9.94E+06	Friedrichskoog	FK1
Haynesina germanica	1.43E+07	Friedrichskoog	FK1

Haynesina germanica	9.41E+06	Friedrichskoog	FK1
Haynesina germanica	1.10E+07	Friedrichskoog	FK1
Haynesina germanica	9.27E+06	Friedrichskoog	FK1
Haynesina germanica	1.28E+07	Friedrichskoog	FK1
Haynesina germanica	9.85E+06	Friedrichskoog	FK1
Haynesina germanica	6.97E+06	Friedrichskoog	FK1
Haynesina germanica	2.00E+07	Friedrichskoog	FK1
Haynesina germanica	1.74E+07	Friedrichskoog	FK1
Haynesina germanica	7.50E+06	Friedrichskoog	FK1
Haynesina germanica	1.65E+07	Friedrichskoog	FK1
Haynesina germanica	1.31E+07	Friedrichskoog	FK1
Haynesina germanica	1.29E+07	Friedrichskoog	FK1
Haynesina germanica	9.61E+06	Friedrichskoog	FK1
Haynesina germanica	8.20E+06	Friedrichskoog	FK1
Haynesina germanica	1.92E+07	Friedrichskoog	FK1
Haynesina germanica	1.54E+07	Friedrichskoog	FK1
Haynesina germanica	1.31E+07	Friedrichskoog	FK1
Haynesina germanica	1.37E+07	Friedrichskoog	FK1
Haynesina germanica	1.02E+07	Friedrichskoog	FK1
Haynesina germanica	1.55E+07	Friedrichskoog	FK1
Haynesina germanica	1.06E+07	Friedrichskoog	FK1
Haynesina germanica	1.56E+07	Friedrichskoog	FK1
Haynesina germanica	1.37E+07	Friedrichskoog	FK1
Haynesina germanica	8.36E+06	Friedrichskoog	FK1
Haynesina germanica	1.57E+07	Friedrichskoog	FK1
Haynesina germanica	9.70E+06	Friedrichskoog	FK1
Haynesina germanica	1.28E+07	Friedrichskoog	FK1
Haynesina germanica	7.89E+06	Friedrichskoog	FK1
Haynesina germanica	2.46E+07	Friedrichskoog	FK1
Haynesina germanica	1.27E+07	Friedrichskoog	FK1
Haynesina germanica	1.12E+07	Friedrichskoog	FK1
Haynesina germanica	9.56E+06	Friedrichskoog	FK1
Haynesina germanica	7.97E+06	Friedrichskoog	FK1
Haynesina germanica	8.33E+06	Friedrichskoog	FK1
Haynesina germanica	1.99E+07	Friedrichskoog	FK1
Haynesina germanica	5.72E+06	Friedrichskoog	FK1
Haynesina germanica	1.00E+07	Friedrichskoog	FK1
Haynesina germanica	5.46E+06	Friedrichskoog	FK1
Haynesina germanica	5.64E+06	Friedrichskoog	FK1
Haynesina germanica	1.43E+07	Friedrichskoog	FK1
Haynesina germanica	1.15E+07	Friedrichskoog	FK1
Haynesina germanica	1.36E+07	Friedrichskoog	FK1
Haynesina germanica	1.23E+07	Friedrichskoog	FK1
Haynesina germanica	1.12E+07	Friedrichskoog	FK1
Haynesina germanica	1.04E+07	Friedrichskoog	FK1
Haynesina germanica	5.00E+06	Friedrichskoog	FK1
Haynesina germanica	9.76E+06	Friedrichskoog	FK1
Haynesina germanica	1.97E+07	Friedrichskoog	FK1
Haynesina germanica	1.19E+07	Friedrichskoog	FK1

Haynesina germanica	1.65E+07	Friedrichskoog	FK1
Haynesina germanica	1.56E+07	Friedrichskoog	FK1
Haynesina germanica	1.49E+07	Friedrichskoog	FK1
Haynesina germanica	2.07E+07	Friedrichskoog	FK1
Haynesina germanica	9.64E+06	Friedrichskoog	FK1
Haynesina germanica	1.06E+07	Friedrichskoog	FK1
Haynesina germanica	1.19E+07	Friedrichskoog	FK1
Haynesina germanica	5.99E+06	Friedrichskoog	FK1
Haynesina germanica	1.49E+07	Friedrichskoog	FK1
Haynesina germanica	2.32E+07	Friedrichskoog	FK1
Haynesina germanica	1.16E+07	Friedrichskoog	FK1
Haynesina germanica	1.31E+07	Friedrichskoog	FK1
Haynesina germanica	1.32E+07	Friedrichskoog	FK1
Haynesina germanica	1.19E+07	Friedrichskoog	FK1
Haynesina germanica	7.47E+06	Friedrichskoog	FK1
Haynesina germanica	1.15E+07	Friedrichskoog	FK1
Haynesina germanica	7.00E+06	Friedrichskoog	FK1
Haynesina germanica	1.54E+07	Friedrichskoog	FK1
Haynesina germanica	1.01E+07	Friedrichskoog	FK1
Haynesina germanica	1.44E+07	Friedrichskoog	FK1
Haynesina germanica	9.10E+06	Friedrichskoog	FK1
Haynesina germanica	9.76E+06	Friedrichskoog	FK1
Haynesina germanica	1.51E+07	Friedrichskoog	FK1
Haynesina germanica	1.23E+07	Friedrichskoog	FK1
Haynesina germanica	1.70E+07	Friedrichskoog	FK1
Haynesina germanica	7.72E+06	Friedrichskoog	FK1
Haynesina germanica	1.32E+07	Friedrichskoog	FK1
Haynesina germanica	8.50E+06	Friedrichskoog	FK1
Haynesina germanica	9.66E+06	Friedrichskoog	FK1
Haynesina germanica	2.40E+07	Friedrichskoog	FK1
Haynesina germanica	1.14E+07	Friedrichskoog	FK1
Haynesina germanica	2.07E+07	Friedrichskoog	FK1
Haynesina germanica	1.25E+07	Friedrichskoog	FK1
Haynesina germanica	8.82E+06	Friedrichskoog	FK1
Haynesina germanica	1.27E+07	Friedrichskoog	FK1
Haynesina germanica	7.75E+06	Friedrichskoog	FK1
Haynesina germanica	1.44E+07	Friedrichskoog	FK1
Haynesina germanica	6.20E+06	Friedrichskoog	FK1
Haynesina germanica	1.02E+07	Friedrichskoog	FK1
Haynesina germanica	1.08E+07	Friedrichskoog	FK1
Haynesina germanica	1.13E+07	Friedrichskoog	FK1
Haynesina germanica	1.66E+07	Friedrichskoog	FK1
Haynesina germanica	1.30E+07	Friedrichskoog	FK1
Haynesina germanica	5.65E+06	Friedrichskoog	FK1
Haynesina germanica	1.23E+07	Friedrichskoog	FK1
Haynesina germanica	9.49E+06	Friedrichskoog	FK1
Haynesina germanica	1.04E+07	Friedrichskoog	FK1
Haynesina germanica	1.20E+07	Friedrichskoog	FK1
Haynesina germanica	1.16E+07	Friedrichskoog	FK1

Haynesina germanica	1.51E+07	Friedrichskoog	FK1
Haynesina germanica	9.97E+06	Friedrichskoog	FK1
Haynesina germanica	7.22E+06	Friedrichskoog	FK1
Haynesina germanica	1.80E+07	Friedrichskoog	FK1
Haynesina germanica	8.34E+06	Friedrichskoog	FK1
Haynesina germanica	1.10E+07	Friedrichskoog	FK1
Haynesina germanica	1.13E+07	Friedrichskoog	FK1
Haynesina germanica	1.16E+07	Friedrichskoog	FK1
Haynesina germanica	6.38E+06	Friedrichskoog	FK1
Haynesina germanica	6.80E+06	Friedrichskoog	FK1
Haynesina germanica	1.36E+07	Friedrichskoog	FK1
Haynesina germanica	4.91E+06	Friedrichskoog	FK1
Haynesina germanica	1.05E+07	Friedrichskoog	FK1
Haynesina germanica	8.21E+06	Friedrichskoog	FK1
Haynesina germanica	6.36E+06	Friedrichskoog	FK1
Haynesina germanica	8.58E+06	Friedrichskoog	FK1
Haynesina germanica	1.31E+07	Friedrichskoog	FK1
Haynesina germanica	1.64E+07	Friedrichskoog	FK1
Haynesina germanica	1.31E+07	Friedrichskoog	FK1
Haynesina germanica	9.47E+06	Friedrichskoog	FK1
Haynesina germanica	7.07E+06	Friedrichskoog	FK1
Haynesina germanica	7.79E+06	Friedrichskoog	FK1
Haynesina germanica	1.45E+07	Friedrichskoog	FK1
Haynesina germanica	4.66E+06	Friedrichskoog	FK1
Haynesina germanica	1.21E+07	Friedrichskoog	FK1
Haynesina germanica	1.51E+07	Friedrichskoog	FK1
Haynesina germanica	1.24E+07	Friedrichskoog	FK1
Haynesina germanica	3.60E+06	Friedrichskoog	FK1
Haynesina germanica	6.29E+06	Friedrichskoog	FK1
Haynesina germanica	7.33E+06	Friedrichskoog	FK1
Haynesina germanica	1.26E+07	Friedrichskoog	FK1
Haynesina germanica	9.01E+06	Friedrichskoog	FK1
Haynesina germanica	1.25E+07	Friedrichskoog	FK1
Haynesina germanica	5.46E+06	Friedrichskoog	FK1
Haynesina germanica	1.85E+07	Friedrichskoog	FK1
Haynesina germanica	1.16E+07	Friedrichskoog	FK1
Haynesina germanica	1.05E+07	Friedrichskoog	FK1
Haynesina germanica	8.40E+06	Friedrichskoog	FK1
Haynesina germanica	1.55E+07	Friedrichskoog	FK1
Haynesina germanica	6.53E+06	Friedrichskoog	FK1
Haynesina germanica	1.50E+07	Friedrichskoog	FK1
Haynesina germanica	6.33E+06	Friedrichskoog	FK1
Haynesina germanica	7.16E+06	Friedrichskoog	FK1
Haynesina germanica	4.82E+06	Friedrichskoog	FK1
Haynesina germanica	7.53E+06	Friedrichskoog	FK1
Haynesina germanica	1.26E+07	Friedrichskoog	FK1
Haynesina germanica	1.30E+07	Friedrichskoog	FK1
Haynesina germanica	6.52E+06	Friedrichskoog	FK1
Haynesina germanica	1.50E+07	Friedrichskoog	FK1

Haynesina germanica	1.17E+07	Friedrichskoog	FK1
Haynesina germanica	8.55E+06	Friedrichskoog	FK1
Hoeglundina elegans	3.45E+08	Rainbow Vent field (MAR)	M176/2 St.24
Hoeglundina elegans	1.08E+08	Rainbow Vent field (MAR)	M176/2 St.24
Hoeglundina elegans	1.26E+08	Rainbow Vent field (MAR)	M176/2 St.24
Hoeglundina elegans	5.22E+07	Rainbow Vent field (MAR)	M176/2 St.24
Hoeglundina elegans	1.09E+08	Rainbow Vent field (MAR)	M176/2 St.24
Hoeglundina elegans	2.80E+07	Rainbow Vent field (MAR)	M176/2 St.24
Labrospira crassimarga	5.59E+07	Bedford Basin	BB March 2022 MC3
Labrospira crassimarga	5.97E+07	Bedford Basin	BB March 2022 MC3
Labrospira crassimarga	3.42E+06	Bedford Basin	BB March 2022 MC3
Rhizammina algaefirmis (fragment)	7.40E+08	Rainbow Vent field (MAR)	M176/2 St.16
Spiroplectammina biformis	2.91E+06	Bedford Basin	BB March 2022 MC3
Spiroplectammina biformis	1.81E+06	Bedford Basin	BB March 2022 MC3
Spiroplectammina biformis	2.37E+06	Bedford Basin	BB March 2022 MC3
Spiroplectammina biformis	2.02E+06	Bedford Basin	BB March 2022 MC3
Spiroplectammina biformis	3.35E+06	Bedford Basin	BB March 2022 MC3
Spiroplectammina biformis	2.36E+06	Bedford Basin	BB March 2022 MC3
Spiroplectammina biformis	1.90E+06	Bedford Basin	BB March 2022 MC3
Spiroplectammina biformis	3.27E+06	Bedford Basin	BB March 2022 MC3
Spiroplectammina biformis	2.22E+06	Bedford Basin	BB March 2022 MC3
Spiroplectammina biformis	2.15E+06	Bedford Basin	BB March 2022 MC3
Spiroplectammina biformis	2.81E+06	Bedford Basin	BB March 2022 MC3
Spiroplectammina biformis	1.71E+06	Bedford Basin	BB March 2022 MC3
Spiroplectammina biformis	1.50E+06	Bedford Basin	BB March 2022 MC3
Spiroplectammina biformis	1.33E+06	Bedford Basin	BB March 2022 MC3
Spiroplectammina biformis	3.19E+06	Bedford Basin	BB March 2022 MC3
Spiroplectammina biformis	2.20E+06	Bedford Basin	BB March 2022 MC3
Spiroplectammina biformis	1.88E+06	Bedford Basin	BB March 2022 MC3

Spiroplectammina biformis	3.58E+06	Bedford Basin	BB March 2022 MC3
Spiroplectammina biformis	1.63E+06	Bedford Basin	BB March 2022 MC3
Spiroplectammina biformis	1.19E+06	Bedford Basin	BB March 2022 MC3
Spiroplectammina biformis	1.55E+06	Bedford Basin	BB March 2022 MC3
Spiroplectammina biformis	8.12E+05	Bedford Basin	BB March 2022 MC3
Spiroplectammina biformis	1.34E+06	Bedford Basin	BB March 2022 MC3
Spiroplectammina biformis	2.87E+06	Bedford Basin	BB March 2022 MC3
Spiroplectammina biformis	2.93E+06	Bedford Basin	BB March 2022 MC3
Spiroplectammina biformis	2.53E+06	Bedford Basin	BB March 2022 MC3
Spiroplectammina biformis	2.16E+06	Bedford Basin	BB March 2022 MC3
Spiroplectammina biformis	2.44E+06	Bedford Basin	BB March 2022 MC3
Spiroplectammina biformis	1.90E+06	Bedford Basin	BB March 2022 MC3
Spiroplectammina biformis	2.58E+06	Bedford Basin	BB March 2022 MC3
Spiroplectammina biformis	1.36E+06	Bedford Basin	BB March 2022 MC3
Spiroplectammina biformis	2.69E+06	Bedford Basin	BB March 2022 MC3
Spiroplectammina biformis	1.53E+06	Bedford Basin	BB March 2022 MC3
Spiroplectammina biformis	1.42E+06	Bedford Basin	BB March 2022 MC3
Spiroplectammina biformis	1.56E+06	Bedford Basin	BB March 2022 MC3
Spiroplectammina biformis	1.38E+06	Bedford Basin	BB March 2022 MC3
Spiroplectammina biformis	1.46E+06	Bedford Basin	BB March 2022 MC3
Spiroplectammina biformis	1.49E+06	Bedford Basin	BB March 2022 MC3
Spiroplectammina biformis	1.85E+06	Bedford Basin	BB March 2022 MC3
Spiroplectammina biformis	3.52E+06	Bedford Basin	BB March 2022 MC3
Spiroplectammina biformis	1.38E+06	Bedford Basin	BB March 2022 MC3
Spiroplectammina biformis	1.89E+06	Bedford Basin	BB March 2022 MC3
Spiroplectammina biformis	1.59E+06	Bedford Basin	BB March 2022 MC3
Spiroplectammina biformis	1.22E+06	Bedford Basin	BB March 2022 MC3
Spiroplectammina biformis	3.25E+06	Bedford Basin	BB March 2022 MC3

Spiroplectammina biformis	1.91E+06	Bedford Basin	BB March 2022 MC3
Spiroplectammina biformis	1.72E+06	Bedford Basin	BB March 2022 MC3
Spiroplectammina biformis	1.72E+06	Bedford Basin	BB March 2022 MC3
Spiroplectammina biformis	1.55E+06	Bedford Basin	BB March 2022 MC3
Spiroplectammina biformis	1.82E+06	Bedford Basin	BB March 2022 MC3
Spiroplectammina biformis	1.33E+06	Bedford Basin	BB March 2022 MC3
Spiroplectammina biformis	2.77E+06	Bedford Basin	BB March 2022 MC3
Spiroplectammina biformis	2.34E+06	Bedford Basin	BB March 2022 MC3
Spiroplectammina biformis	1.51E+06	Bedford Basin	BB March 2022 MC3
Spiroplectammina biformis	1.54E+06	Bedford Basin	BB March 2022 MC3
Spiroplectammina biformis	2.77E+06	Bedford Basin	BB March 2022 MC3
Spiroplectammina biformis	1.90E+06	Bedford Basin	BB March 2022 MC3
Spiroplectammina biformis	1.83E+06	Bedford Basin	BB March 2022 MC3
Spiroplectammina biformis	1.83E+06	Bedford Basin	BB March 2022 MC3
Stainforthia fusiformis	6.21E+05	Bedford Basin	BB March 2022 MC1
Stainforthia fusiformis	9.80E+05	Bedford Basin	BB March 2022 MC1
Stainforthia fusiformis	8.37E+05	Bedford Basin	BB March 2022 MC1
Stainforthia fusiformis	5.00E+05	Bedford Basin	BB March 2022 MC1
Stainforthia fusiformis	5.14E+05	Bedford Basin	BB March 2022 MC1
Stainforthia fusiformis	4.53E+05	Bedford Basin	BB March 2022 MC1
Stainforthia fusiformis	4.69E+05	Bedford Basin	BB March 2022 MC1
Stainforthia fusiformis	3.88E+05	Bedford Basin	BB March 2022 MC1
Stainforthia fusiformis	8.68E+05	Bedford Basin	BB March 2022 MC1
Stainforthia fusiformis	6.53E+05	Bedford Basin	BB March 2022 MC2
Stainforthia fusiformis	1.28E+06	Bedford Basin	BB March 2022 MC2
Stainforthia fusiformis	8.09E+05	Bedford Basin	BB March 2022 MC2
Stainforthia fusiformis	9.37E+05	Bedford Basin	BB March 2022 MC2
Stainforthia fusiformis	9.55E+05	Bedford Basin	BB March 2022 MC2
Stainforthia fusiformis	7.10E+05	Bedford Basin	BB March 2022 MC2
Stainforthia fusiformis	6.11E+05	Bedford Basin	BB March 2022 MC2
Stainforthia fusiformis	1.11E+06	Bedford Basin	BB March 2022 MC2
Stainforthia fusiformis	3.51E+05	Bedford Basin	BB March 2022 MC2
Stainforthia fusiformis	1.32E+06	Bedford Basin	BB March 2022 MC2
Stainforthia fusiformis	6.86E+05	Bedford Basin	BB March 2022 MC2
Stainforthia fusiformis	4.41E+05	Bedford Basin	BB March 2022 MC2
Stainforthia fusiformis	5.69E+05	Bedford Basin	BB March 2022 MC2
Stainforthia fusiformis	6.31E+05	Bedford Basin	BB March 2022 MC2
Stainforthia fusiformis	4.29E+05	Bedford Basin	BB March 2022 MC2
Stainforthia fusiformis	9.87E+05	Bedford Basin	BB March 2022 MC2

Stainforthia fusiformis	8.43E+05	Bedford Basin	BB March 2022 MC2
Stainforthia fusiformis	1.19E+06	Bedford Basin	BB March 2022 MC2
Stainforthia fusiformis	8.96E+05	Bedford Basin	BB March 2022 MC2
Stainforthia fusiformis	1.98E+06	Bedford Basin	BB March 2022 MC2
Stainforthia fusiformis	1.02E+06	Bedford Basin	BB March 2022 MC2
Stainforthia fusiformis	1.13E+06	Bedford Basin	BB March 2022 MC2
Stainforthia fusiformis	9.15E+05	Bedford Basin	BB March 2022 MC2
Stainforthia fusiformis	6.30E+05	Bedford Basin	BB March 2022 MC2
Stainforthia fusiformis	3.61E+05	Bedford Basin	BB March 2022 MC2
Stainforthia fusiformis	7.32E+05	Bedford Basin	BB March 2022 MC2
Stainforthia fusiformis	9.84E+05	Bedford Basin	BB March 2022 MC2
Stainforthia fusiformis	5.19E+05	Bedford Basin	BB March 2022 MC2
Stainforthia fusiformis	1.09E+06	Bedford Basin	BB March 2022 MC2
Stainforthia fusiformis	8.06E+05	Bedford Basin	BB March 2022 MC2
Stainforthia fusiformis	8.66E+05	Bedford Basin	BB March 2022 MC2
Stainforthia fusiformis	4.21E+05	Bedford Basin	BB March 2022 MC2
Stainforthia fusiformis	9.20E+05	Bedford Basin	BB March 2022 MC2
Stainforthia fusiformis	6.10E+05	Bedford Basin	BB March 2022 MC2
Stainforthia fusiformis	1.15E+06	Bedford Basin	BB March 2022 MC2
Stainforthia fusiformis	8.25E+05	Bedford Basin	BB March 2022 MC2
Stainforthia fusiformis	7.76E+05	Bedford Basin	BB March 2022 MC2
Stainforthia fusiformis	7.44E+05	Bedford Basin	BB March 2022 MC2
Stainforthia fusiformis	6.35E+05	Bedford Basin	BB March 2022 MC2
Stainforthia fusiformis	8.59E+05	Bedford Basin	BB March 2022 MC2
Stainforthia fusiformis	8.03E+05	Bedford Basin	BB March 2022 MC2
Stainforthia fusiformis	6.75E+05	Bedford Basin	BB March 2022 MC2
Stainforthia fusiformis	6.55E+05	Bedford Basin	BB March 2022 MC2
Stainforthia fusiformis	1.09E+06	Bedford Basin	BB March 2022 MC2
Stainforthia fusiformis	1.14E+06	Bedford Basin	BB March 2022 MC2
Stainforthia fusiformis	4.22E+05	Bedford Basin	BB March 2022 MC2
Stainforthia fusiformis	1.05E+06	Bedford Basin	BB March 2022 MC2
Stainforthia fusiformis	6.29E+05	Bedford Basin	BB March 2022 MC2
Stainforthia fusiformis	1.19E+06	Bedford Basin	BB March 2022 MC2
Stainforthia fusiformis	9.99E+05	Bedford Basin	BB March 2022 MC2
Stainforthia fusiformis	4.19E+05	Bedford Basin	BB March 2022 MC2
Stainforthia fusiformis	1.34E+06	Bedford Basin	BB March 2022 MC2
Stainforthia fusiformis	6.28E+05	Bedford Basin	BB March 2022 MC2
Stainforthia fusiformis	9.22E+05	Bedford Basin	BB March 2022 MC2
Stainforthia fusiformis	7.60E+05	Bedford Basin	BB March 2022 MC2
Stainforthia fusiformis	5.93E+05	Bedford Basin	BB March 2022 MC2
Stainforthia fusiformis	1.33E+06	Bedford Basin	BB March 2022 MC2
Stainforthia fusiformis	2.59E+05	Bedford Basin	BB March 2022 MC2
Stainforthia fusiformis	8.24E+05	Bedford Basin	BB March 2022 MC2
Stainforthia fusiformis	4.92E+05	Bedford Basin	BB March 2022 MC2
Stainforthia fusiformis	6.37E+05	Bedford Basin	BB March 2022 MC2
Stainforthia fusiformis	9.28E+05	Bedford Basin	BB March 2022 MC2
Stainforthia fusiformis	7.12E+05	Bedford Basin	BB March 2022 MC2
Stainforthia fusiformis	8.71E+05	Bedford Basin	BB March 2022 MC2
Stainforthia fusiformis	8.66E+05	Bedford Basin	BB March 2022 MC2

Stainforthia fusiformis	1.14E+06	Bedford Basin	BB March 2022 MC2
Stainforthia fusiformis	1.03E+06	Bedford Basin	BB March 2022 MC2
Stainforthia fusiformis	7.18E+05	Bedford Basin	BB March 2022 MC2
Stainforthia fusiformis	5.73E+05	Bedford Basin	BB March 2022 MC2
Stainforthia fusiformis	8.94E+05	Bedford Basin	BB March 2022 MC2
Stainforthia fusiformis	8.09E+05	Bedford Basin	BB March 2022 MC2
Stainforthia fusiformis	2.89E+05	Bedford Basin	BB March 2022 MC2
Stainforthia fusiformis	1.10E+06	Bedford Basin	BB March 2022 MC2
Stainforthia fusiformis	1.54E+06	Bedford Basin	BB March 2022 MC2
Stainforthia fusiformis	6.14E+05	Bedford Basin	BB March 2022 MC2
Stainforthia fusiformis	5.81E+05	Bedford Basin	BB March 2022 MC2
Stainforthia fusiformis	5.36E+05	Bedford Basin	BB March 2022 MC2
Stainforthia fusiformis	4.94E+05	Bedford Basin	BB March 2022 MC2
Stainforthia fusiformis	7.96E+05	Bedford Basin	BB March 2022 MC2
Stainforthia fusiformis	6.91E+05	Bedford Basin	BB March 2022 MC2
Stainforthia fusiformis	1.23E+06	Bedford Basin	BB March 2022 MC2
Stainforthia fusiformis	7.28E+05	Bedford Basin	BB March 2022 MC2
Stainforthia fusiformis	9.59E+05	Bedford Basin	BB March 2022 MC2
Stainforthia fusiformis	7.99E+05	Bedford Basin	BB March 2022 MC2
Stainforthia fusiformis	5.89E+05	Bedford Basin	BB March 2022 MC2
Stainforthia fusiformis	6.34E+05	Bedford Basin	BB March 2022 MC2
Stainforthia fusiformis	6.82E+05	Bedford Basin	BB March 2022 MC2
Stainforthia fusiformis	5.52E+05	Bedford Basin	BB March 2022 MC2
Stainforthia fusiformis	3.69E+05	Bedford Basin	BB March 2022 MC2
Stainforthia fusiformis	1.13E+06	Bedford Basin	BB March 2022 MC2
Stainforthia fusiformis	6.41E+05	Bedford Basin	BB March 2022 MC2
Stainforthia fusiformis	1.14E+06	Bedford Basin	BB March 2022 MC2
Stainforthia fusiformis	1.44E+06	Bedford Basin	BB March 2022 MC2
Stainforthia fusiformis	5.72E+05	Bedford Basin	BB March 2022 MC2
Stainforthia fusiformis	6.11E+05	Bedford Basin	BB March 2022 MC2
Stainforthia fusiformis	6.51E+05	Bedford Basin	BB March 2022 MC2
Stainforthia fusiformis	7.28E+05	Bedford Basin	BB March 2022 MC2
Stainforthia fusiformis	7.54E+05	Bedford Basin	BB March 2022 MC2
Stainforthia fusiformis	3.86E+05	Bedford Basin	BB March 2022 MC2
Stainforthia fusiformis	4.79E+05	Bedford Basin	BB March 2022 MC2
Stainforthia fusiformis	9.00E+05	Bedford Basin	BB March 2022 MC2
Stainforthia fusiformis	1.01E+06	Bedford Basin	BB March 2022 MC2
Stainforthia fusiformis	8.87E+05	Bedford Basin	BB March 2022 MC2
Stainforthia fusiformis	7.01E+05	Bedford Basin	BB March 2022 MC2
Stainforthia fusiformis	5.01E+05	Bedford Basin	BB March 2022 MC2
Stainforthia fusiformis	6.09E+05	Bedford Basin	BB March 2022 MC2
Stainforthia fusiformis	8.29E+05	Bedford Basin	BB March 2022 MC2
Stainforthia fusiformis	9.13E+05	Bedford Basin	BB March 2022 MC2
Stainforthia fusiformis	3.86E+05	Bedford Basin	BB March 2022 MC2
Stainforthia fusiformis	6.58E+05	Bedford Basin	BB March 2022 MC2
Stainforthia fusiformis	1.18E+06	Bedford Basin	BB March 2022 MC2
Stainforthia fusiformis	9.45E+05	Bedford Basin	BB March 2022 MC2
Stainforthia fusiformis	7.57E+05	Bedford Basin	BB March 2022 MC2
Stainforthia fusiformis	7.78E+05	Bedford Basin	BB March 2022 MC2

Stainforthia fusiformis	1.03E+06	Bedford Basin	BB March 2022 MC2
Stainforthia fusiformis	9.64E+05	Bedford Basin	BB March 2022 MC2
Stainforthia fusiformis	9.88E+05	Bedford Basin	BB March 2022 MC2
Stainforthia fusiformis	7.96E+05	Bedford Basin	BB March 2022 MC2
Stainforthia fusiformis	7.48E+05	Bedford Basin	BB March 2022 MC2
Stainforthia fusiformis	1.02E+06	Bedford Basin	BB March 2022 MC2
Stainforthia fusiformis	8.65E+05	Bedford Basin	BB March 2022 MC2
Stainforthia fusiformis	7.67E+05	Bedford Basin	BB March 2022 MC2
Stainforthia fusiformis	9.49E+05	Bedford Basin	BB March 2022 MC2
Stainforthia fusiformis	7.81E+05	Bedford Basin	BB March 2022 MC2
Stainforthia fusiformis	1.05E+06	Bedford Basin	BB March 2022 MC2
Stainforthia fusiformis	1.02E+06	Bedford Basin	BB March 2022 MC2
Uvigerina akitaensis	2.21E+07	Sagami Bay	NSB 2019
Uvigerina akitaensis	7.71E+06	Sagami Bay	NSB 2019
Uvigerina akitaensis	1.18E+07	Sagami Bay	NSB 2019
Uvigerina akitaensis	2.16E+07	Sagami Bay	NSB 2019
Uvigerina akitaensis	1.30E+07	Sagami Bay	NSB 2019
Uvigerina akitaensis	1.51E+07	Sagami Bay	NSB 2019
Uvigerina akitaensis	2.68E+07	Sagami Bay	NSB 2019
Uvigerina akitaensis	1.31E+07	Sagami Bay	NSB 2019
Uvigerina akitaensis	1.72E+07	Sagami Bay	NSB 2019
Uvigerina akitaensis	7.16E+06	Sagami Bay	NSB 2019
Uvigerina akitaensis	1.16E+07	Sagami Bay	NSB 2019
Uvigerina akitaensis	2.32E+07	Sagami Bay	NSB 2019
Uvigerina akitaensis	3.20E+07	Sagami Bay	NSB 2019
Uvigerina akitaensis	1.97E+07	Sagami Bay	NSB 2019
Uvigerina akitaensis	6.38E+06	Sagami Bay	NSB 2019
Uvigerina akitaensis	2.91E+07	Sagami Bay	NSB 2019
Uvigerina akitaensis	6.54E+06	Sagami Bay	NSB 2019
Uvigerina akitaensis	8.60E+06	Sagami Bay	NSB 2019
Uvigerina akitaensis	8.65E+06	Sagami Bay	NSB 2019
Uvigerina akitaensis	1.81E+07	Sagami Bay	NSB 2019
Uvigerina akitaensis	1.53E+07	Sagami Bay	NSB 2019
Uvigerina akitaensis	1.41E+07	Sagami Bay	NSB 2019
Uvigerina akitaensis	5.62E+06	Sagami Bay	NSB 2019
Uvigerina akitaensis	4.57E+06	Sagami Bay	NSB 2019

Tab.ST6: Total benthic foraminiferal phosphate storage in foraminiferal assemblages from the Southern North Sea. For each station the coordinates as well as the reference to the original publication of the assemblage data is given.

Station	Longitude [°E]	Latitude [°N]	Foraminiferal phosphate [g/m2]	Reference for assemblage data
FK2	8.83618	54.03019	0.16300	This study
Schobüll, tidal flat	8.98972	54.51254	0.04009	[26]
Tümlau D15	8.67531	54.36738	0.00020	[26,27]
Tümlau E11	8.6776	54.36776	0.00003	[26,27]
Tümlau E12	8.67762	54.36761	0.00026	[26,27]
Tümlau F13	8.67667	54.36457	0.00006	[26,27]
Tümlau F14	8.6763	54.3645	0.00040	[26,27]
Bunkerpier	7.88842	54.17843	0.05037	[26]
dNooj1	4.5	53.65	0.02637	[28]
dNooj2	4.5	53.65	0.02035	[28]
dNooj3	4.5	53.75	0.01983	[28]
dNooj4	4.5	53.75	0.01205	[28]
dNooj5	4.5	53.9	0.00436	[28]
dNooj6	4.5	53.9	0.00429	[28]
dNooj7	4.5	54	0.00230	[28]
dNooj8	4.5	54	0.01452	[28]
dNooj9	4.5	53.65	0.02889	[28]
dNooj10	4.5	53.65	0.03330	[28]
dNooj11	4.5	53.83333	0.00478	[28]
dNooj12	4.5	53.83333	0.00566	[28]
dNooj13	4.5	53.9	0.00197	[28]
dNooj14	4.5	53.9	0.00247	[28]
dNooj15	4.5	54	0.00438	[28]
dNooj16	4.5	54	0.00372	[28]
dNooj17	4.5	53.5	0.00042	[28]
dNooj18	4.5	53.65	0.00789	[28]
dNooj19	4.5	53.65	0.00866	[28]
dNooj20	4.5	54	0.00338	[28]
dNooj21	4.5	54	0.00281	[28]
dNooj22	4.5	54	0.00011	[28]
dNooj23	4.5	53.36666	0.00019	[28]
dNooj24	4.5	53.7	0.01330	[28]
dNooj25	4.5	53.7	0.00881	[28]
Scheldt 1	3.7085	51.354	0.01383	[29]
Scheldt 2	3.7085	51.354	0.00901	[29]
Phleg I 1	4.071	51.985	0.00023	[30]
Phleg I 3	4.071	51.985	0.00020	[30]
Phleg I 6	4.071	51.985	0.00046	[30]
Phleg I 8	4.071	51.985	0.00086	[30]
Phleg I 11	4.071	51.985	0	[30]
Phleg I 14	4.071	51.985	0	[30]
Phleg III 31	4.13	51.401	0	[30]
Phleg III 32	4.13	51.401	0	[30]
Phleg III 34	4.13	51.401	0.00017	[30]

Phleg III 35	4.13	51.401	0.04319	30
Phleg III 36	4.13	51.401	0	30
Phleg III 37	4.13	51.401	0.00005	30
Phleg III 38	4.13	51.401	0.00005	30
Phleg IV 39	4.283	51.468	0.01366	30
Phleg IV 41	4.283	51.468	0.00482	30
Phleg IV 44	4.283	51.468	0.00453	30
Phleg IV 47	4.283	51.468	0.00105	30
Phleg IV 48	4.283	51.468	0.00048	30
Phleg IV 49	4.283	51.468	0	30
Phleg V 51	4.263	51.337	0	30
Phleg V 52	4.263	51.337	0.00024	30
Phleg V 53	4.263	51.337	0	30
Phleg V 54	4.263	51.337	0	30
Phleg V 55	4.263	51.337	0	30
Phleg V 56	4.263	51.337	0	30
Phleg V 59	4.263	51.337	0	30
Phleg VI 60	4.142	51.44	0.00061	30
Phleg VI 61	4.142	51.44	0	30
Phleg VI 64	4.142	51.44	0	30
Phleg VI 66	4.142	51.44	0.00116	30
Phleg VI 69	4.142	51.44	0.00481	30
Phleg VI 71	4.142	51.44	0.03598	30
Phleg VI 72	4.142	51.44	0.00234	30
Phleg VI 74	4.142	51.44	0.00016	30
Phleg VI 76	4.142	51.44	0.00067	30
Phleg VI 77	4.142	51.44	0.00055	30
Phleg VII 78	4.237	51.384	0	30
Phleg VII 81	4.237	51.384	0.00082	30
Phleg VII 84	4.237	51.384	0.00015	30
Phleg VII 86	4.237	51.384	0.00027	30
Phleg VIII 90	3.681	51.447	0.00028	30
Phleg VIII 91	3.681	51.447	0	30
Phleg VIII 93	3.681	51.447	0.00015	30
Phleg VIII 94	3.681	51.447	0.02629	30
Phleg VIII 95	3.681	51.447	0.00021	30
Phleg VIII 96	3.681	51.447	0.00157	30
Phleg VIII 97	3.681	51.447	0	30
Phleg VIII 98	3.681	51.447	0	30
Phleg IX 99	3.86	51.789	0.00278	30
Phleg IX 101	3.86	51.789	0.00056	30
Phleg IX 102	3.86	51.789	0.00036	30
Phleg IX 103	3.86	51.789	0.00000	30
Phleg IX 104	3.86	51.789	0.00035	30
Phleg IX 105	3.86	51.789	0.00063	30
Phleg IX 106	3.86	51.789	0.02586	30
Phleg IX 110	3.86	51.789	0.00046	30
Phleg IX 111	3.86	51.789	0.00153	30
Phleg IX 112	3.86	51.789	0.00329	30

Phleg IX 114	3.86	51.789	0.00022	30
Phleg IX 115	3.86	51.789	0.00003	30
Phleg IX 117	3.86	51.789	0.0002	30
Phleg IX 118	3.86	51.789	0.00707	30
Mood1	4.5	53.43333	0.00133	31
Mood2	4.5	53.43333	0.00012	31
Mood3	4.5	53.62	0.0161	31
Mood4	4.5	53.62	0.00804	31
Mood5	4.5	53.73	0.01852	31
Mood6	4.5	53.73	0.00439	31
Mood7	4.5	54	0.00668	31
Murr1	4.58867	53.6157	0.02135	32
Murr2	4.634	53.625	0.00256	32
Murr3	4.634	54.1125	0.00008	32
Murr4	4.634	54.629	0.00009	32
Murr5	5.6134	54.059	0.00043	32
Murr6	6.433	54.619	0.00035	32
Murr7	7.02	54.1125	0.00367	32
Murr8	7.4948	54.209	0.00099	32
JapsandA1	8.46	54.57	0	26
JapsandA2	8.46	54.57	0	26
Japsand1	8.46	54.57	0	26
JapsandB1	8.46	54.57	0.01	26
JapsandB2	8.46	54.57	0	26
Japsand2	8.46	54.57	0	26
JapsandF1	8.48	54.58	0	26
JapsandF2	8.48	54.58	0	26
Japsand3	8.47	54.57	0	26
Japsand4	8.47	54.57	0	26
Japsand5	8.47	54.57	0	26
Japsand6	8.47	54.57	0	26
Japsand7	8.47	54.57	0	26
JapsandE1	8.48	54.57	0	26
JapsandE2	8.48	54.57	0	26
JapsandC1	8.49	54.57	0	26
JapsandC2	8.49	54.57	0	26
Japsand8	8.49	54.56	0	26
JapsandD1	8.5	54.56	0	26
JapsandD2	8.5	54.56	0	26
JapsandG1	8.51	54.56	0	26
JapsandG2	8.51	54.56	0	26
Hooge	8.52	54.57	0	26

Tab.ST7: Total benthic foraminiferal phosphate storage in foraminiferal assemblages from 10 – 15°S off Peru. For each station the coordinates as well as the reference to the original publication of the assemblage data is given.

Station	Longitude [°W]	Latitude [°S]	Foraminiferal phosphate (g/m^2)	Reference for assemblage data
M137 – 681 MUC 13	282.8205	12.22517	0.05225	[33]
M137 – 641 MUC 7	282.75018	12.278	0.038	[33]
M137 – 695 MUC 17	282.75034	12.27967	0.0456	[33]
M137 – 608 MUC 4	282.59534	12.38767	0.00095	[33]
M137 – 776 MUC 32	282.56183	12.415	0.05035	[33]
M137 – 788 MUC 34	282.51166	12.415	0.3306	[33]
M137 – 735 MUC 24	282.65433	12.415	0.1444	[33]
M137 – 670 MUC 12	282.41666	12.415	0.0133	[33]
M77/1 -540 MUC 49	282.20999	11.00017	0.0095	[33]
M77/1-583 MUC 65	281.94901	11.11433	0.0437	[33]
M77/1-473 MUC 32	281.83435	11.00017	0.057	[33]
M77-1-449 MUC 19	281.83383	11.00017	0.04465	[33]
M77/1-456 MUC 22	281.6795	11.00017	0.02375	[33]
M77/1-459 MUC 25	281.57333	11.0005	0.00285	[33]
Card01	282.78217	12.03167	0.00292	[34]
Card02	282.71216	12.046	0.00259	[34]
Card03	282.62451	12.039	0.00402	[34]
Card04	282.51651	12.04883	0.00360	[34]
Card05	282.34882	12.037	0.00370	[34]
Card06	282.78217	12.03167	0.00251	[34]
Card07	282.71216	12.046	0.00258	[34]
Card08	282.51651	12.04883	0.00547	[34]
Card09	282.34882	12.037	0.00846	[34]
Card10	282.78217	12.03167	0.00610	[34]
Card11	282.71216	12.046	0.00459	[34]
Card12	282.62451	12.039	0.00684	[34]
Card13	282.51651	12.04883	0.00998	[34]
Card14	282.34882	12.037	0.00348	[34]
Card15	283.68701	14.02	0.00640	[34]
Card16	283.58002	14.072	0.01354	[34]
Card17	283.491	14.125	0.02733	[34]
Card18	282.78217	12.03167	0.01880	[34]
Card19	282.71216	12.046	0.01654	[34]
Card20	282.34882	12.037	0.08890	[34]
Card21	283.58002	14.072	0.00811	[34]

Supplementary references

1. Clarke, K. *et al.* The β/α Peak Height Ratio of ATP: A MEASURE OF FREE [Mg2+] USING 31P NMR*. *J. Biol. Chem.* **271**, 21142–21150 (1996).

2. Müller, W. E. G., Schröder, H. C. & Wang, X. Inorganic Polyphosphates As Storage for and Generator of Metabolic Energy in the Extracellular Matrix. *Chem. Rev.* **119**, 12337–12374 (2019).

3. Müller, W. E. G. *et al.* Nanoparticle-directed and ionically forced polyphosphate coacervation: a versatile and reversible core–shell system for drug delivery. *Sci. Rep.* **10**, 17147 (2020).

4. Grabherr, M. G. *et al.* Full-length transcriptome assembly from RNA-Seq data without a reference genome. *Nat. Biotechnol.* **29**, 644–652 (2011).

5. Altschul, S. F. *et al.* Gapped BLAST and PSI-BLAST: a new generation of protein database search programs. *Nucleic Acids Res.* **25**, 3389–3402 (1997).

6. Rice, P., Longden, I. & Bleasby, A. EMBOSS: The European Molecular Biology Open Software Suite. *Trends Genet.* **16**, 276–277 (2000).

7. Katoh, K. & Standley, D. M. MAFFT Multiple Sequence Alignment Software Version 7: Improvements in Performance and Usability. *Mol. Biol. Evol.* **30**, 772–780 (2013).

8. Nguyen, L.-T., Schmidt, H. A., von Haeseler, A. & Minh, B. Q. IQ-TREE: A Fast and Effective Stochastic Algorithm for Estimating Maximum-Likelihood Phylogenies. *Mol. Biol. Evol.* **32**, 268–274 (2015).

9. Hayward, B. *et al.* Molecular and morphological taxonomy of living Ammonia and related taxa (Foraminifera) and their biogeography. *Micropaleontology* **67**, 109–313 (2021).

10. Capella-Gutiérrez, S., Silla-Martínez, J. M. & Gabaldón, T. trimAl: a tool for automated alignment trimming in large-scale phylogenetic analyses. *Bioinformatics* **25**, 1972–1973 (2009).

11. Soubrier, J. *et al.* The Influence of Rate Heterogeneity among Sites on the Time Dependence of Molecular Rates. *Mol. Biol. Evol.* **29**, 3345–3358 (2012).

12. Kalyaanamoorthy, S., Minh, B. Q., Wong, T. K. F., von Haeseler, A. & Jermiin, L. S. ModelFinder: fast model selection for accurate phylogenetic estimates. *Nat. Methods* **14**, 587–589 (2017).

13. Hoang, D. T., Chernomor, O., von Haeseler, A., Minh, B. Q. & Vinh, L. S. UFBoot2: Improving the Ultrafast Bootstrap Approximation. *Mol. Biol. Evol.* **35**, 518–522 (2018).

14. Guindon, S. *et al.* New Algorithms and Methods to Estimate Maximum-Likelihood Phylogenies: Assessing the Performance of PhyML 3.0. *Syst. Biol.* **59**, 307–321 (2010).

15. Wadden Sea World Natural Heritage. https://www.bmuv.de/en/topics/nature-and-biological-diversity/overview-nature-and-biological-diversity/overview-international-biological-diversity/unesco-world-heritage-convention/wadden-sea-world-natural-heritage.

16. Pätsch, J. & Lenhart, H. J. Daily Loads of Nutrients, Total Alkalinity, Dissolved Inorganic Carbon and Dissolved Organic Carbon of the European continental Rivers

for the Years 1977-2002. (Updated to 2019). *Berichte aus dem Zent. für Meeres- und Klimaforschung. R. B Ozeanogr.* **48**, (2004).

17. Beusen, A. H. W. *et al.* Exploring river nitrogen and phosphorus loading and export to global coastal waters in the Shared Socio-economic pathways. *Glob. Environ. Chang.* **72**, 102426 (2022).

18. Schlitzer, R. Ocean Data View. (2015).

19. Glud, R. N. *et al.* In situ microscale variation in distribution and consumption of 2: A case study from a deep ocean margin sediment (Sagami Bay, Japan). *Limnol. Oceanogr.* **54**, 1–12 (2009).

20. Jansen, S. *et al.* Functioning of intertidal flats inferred from temporal and spatial dynamics of O2, H2S and pH in their surface sediment. *Ocean Dyn. 2009 592* **59**, 317–332 (2009).

21. Rakshit, S., Dale, A. W., Wallace, D. W. & Algar, C. K. Sources and sinks of bottom water oxygen in a seasonally hypoxic fjord. *Front. Mar. Sci.* **10**, (2023).

22. Glock, N. *et al.* Evironmental influences on the pore density of Bolivina spissa (Cushman). *J. Foraminifer. Res.* **41**, 22–32 (2011).

23. Pfannkuche, O., Frank, M., Schneider, R. & Stramma, L. *Climate-biogeochemistry interactions in the tropical ocean of the SE-American oxygen minimum zone – Cruise No. M77 – October 22, 2008 – February 18, 2009 – Talcahuano (Chile) – Colon (Panama).* (2011). doi:10.2312/cr_m77.

24. Haalboom, S. & de Stigter, H. Patterns of (trace) metals and microorganisms in the Rainbow hydrothermal vent plume at the Mid-Atlantic Ridge. doi:doi:10.25850/nioz/7b.b.s.

25. Ziebis, W. *et al.* Interstitial fluid chemistry of sediments underlying the North Atlantic gyre and the influence of subsurface fluid flow. *Earth Planet. Sci. Lett.* **323–324**, 79–91 (2012).

26. Schmidt, S. & Schönfeld, J. Living and dead foraminiferal assemblage from the supratidal sand Japsand, North Frisian Wadden Sea: distributional patterns and controlling factors. *Helgol. Mar. Res. 2021 751* **75**, 1–22 (2021).

27. Müller-Navarra, K., Milker, Y. & Schmiedl, G. Natural and anthropogenic influence on the distribution of salt marsh foraminifera in the Bay of Tümlau, German North Sea. *J. Foraminifer. Res.* **46**, 61–74 (2016).

28. de Nooijer, L. J., Duijnstee, I. A. P., Bergman, M. J. N. & van der Zwaan, G. J. The ecology of benthic foraminifera across the Frisian Front, southern North Sea. *Estuar. Coast. Shelf Sci.* **78**, 715–726 (2008).

29. Brouwer, M. G. M. *et al.* Differential response of intertidal foraminifera to community recovery following experimentally induced hypoxia. *J. Foraminifer. Res.* **45**, 220–234 (2015).

30. Phleger, F. B. Foraminiferal populations and marine marsh processes. *Limnol. Oceanogr.* **15**, 522–534 (1970).

31. Moodley, L. Southern North Sea seafloor and subsurface distribution of living benthic foraminifera. *Netherlands J. Sea Res.* **27**, 57–71 (1990).

32. Murray, J. W. Distribution and population dynamics of benthic foraminifera from the southern North Sea. *J. Foraminifer. Res.* **22**, 114–128 (1992).

33. Glock, N. *et al.* A hidden sedimentary phosphate pool inside benthic foraminifera from the Peruvian upwelling region might nucleate phosphogenesis. *Geochim. Cosmochim. Acta* **289**, 14–32 (2020).

34. Cardich, J. *et al.* Calcareous benthic foraminifera from the upper central Peruvian margin: control of the assemblage by pore water redox and sedimentary organic matter. *Mar. Ecol. Prog. Ser.* **535**, 63–87 (2015).
