# Supplementary material for: Widespread occurrence and relevance of phosphate storage in foraminifera
Source: Nature. 2025 Jan 15;638(8052):1000–6. doi: 10.1038/s41586-024-08431-8 (PMC11864969; doi:10.1038/s41586-024-08431-8)
Supplement: Supplementary file 2 — Reporting Summary [file 41586_2024_8431_MOESM2_ESM.pdf]

nature portfolio

Corresponding author(s): Nicolaas Glock

Last updated by author(s): Oct 11, 2024

Reporting Summary

Nature Portfolio wishes to improve the reproducibility of the work that we publish. This form provides structure for consistency and transparency in reporting. For further information on Nature Portfolio policies, see our Editorial Policies and the Editorial Policy Checklist.

Statistics

For all statistical analyses, confirm that the following items are present in the figure legend, table legend, main text, or Methods section.

n/a	Confirmed	
☐	☒	The exact sample size (*n*) for each experimental group/condition, given as a discrete number and unit of measurement
☐	☒	A statement on whether measurements were taken from distinct samples or whether the same sample was measured repeatedly
☒	☐	The statistical test(s) used AND whether they are one- or two-sided<br>*Only common tests should be described solely by name; describe more complex techniques in the Methods section.*
☒	☐	A description of all covariates tested
☒	☐	A description of any assumptions or corrections, such as tests of normality and adjustment for multiple comparisons
☐	☒	A full description of the statistical parameters including central tendency (e.g. means) or other basic estimates (e.g. regression coefficient) AND variation (e.g. standard deviation) or associated estimates of uncertainty (e.g. confidence intervals)
☒	☐	For null hypothesis testing, the test statistic (e.g. *F*, *t*, *r*) with confidence intervals, effect sizes, degrees of freedom and *P* value noted<br>*Give P values as exact values whenever suitable.*
☒	☐	For Bayesian analysis, information on the choice of priors and Markov chain Monte Carlo settings
☒	☐	For hierarchical and complex designs, identification of the appropriate level for tests and full reporting of outcomes
☒	☐	Estimates of effect sizes (e.g. Cohen's *d*, Pearson's *r*), indicating how they were calculated

Our web collection on statistics for biologists contains articles on many of the points above.

Software and code

Policy information about availability of computer code

Data collection

NMR spectroscopy: Data was acquired, using TopSpin version 3.6.4 and all all spectra were processed utilizing Topspin Version 4.1.4.
cryo-SEM/EDS: EDS data was acquired using TeamEDS, version 4.6.0052.0238.
Geographic maps were created using Ocean Data View (ODV) version 5.5.1.
Protein homologs in publicly available protein sequences were identified using the KEGG KAAS tool (https://doi.org/10.1093/nar/gkm321)
Raw reads for metabarcoding were quality-filtered with the FASTX-Toolkit 0.0.13
For the transcriptome assembly for Ammonia veneta the Trinity assembly tool (Version 2.15.1) was used.

Data analysis

NMR spectroscopy: Data was acquired, using TopSpin version 3.6.4 and all all spectra were processed utilizing Topspin Version 4.1.4.
cryo-SEM/EDS: EDS data was acquired using TeamEDS, version 4.6.0052.0238.
Geographic maps were created using Ocean Data View (ODV) version 5.5.1.
Protein homologs in publicly available protein sequences were identified using the KEGG KAAS tool (https://doi.org/10.1093/nar/gkm321)
Raw reads for metabarcoding were quality-filtered with the FASTX-Toolkit 0.0.13
For the transcriptome assembly for Ammonia veneta the Trinity assembly tool (Version 2.15.1) was used.

For manuscripts utilizing custom algorithms or software that are central to the research but not yet described in published literature, software must be made available to editors and reviewers. We strongly encourage code deposition in a community repository (e.g. GitHub). See the Nature Portfolio guidelines for submitting code & software for further information.

Data

Policy information about availability of data

All manuscripts must include a data availability statement. This statement should provide the following information, where applicable:

- Accession codes, unique identifiers, or web links for publicly available datasets
- A description of any restrictions on data availability
- For clinical datasets or third party data, please ensure that the statement adheres to our policy

Publicly available protein sequences and transcriptomes were downloaded from NCBI database (https://www.ncbi.nlm.nih.gov/) via following accessions: GIDR00000000.1 (Ammonia confertitesta) and GIHI00000000.1 (Globobulimina pacifica) and GCA_000512085.1 (Reticulomyxa filosa). Raw data for the transcriptome assembly of Ammonia veneta was obtained from the Sequence Read Archive (SRR18700766). Accessions (NCBI and KEGG databases) for the individual creatine kinase sequences used are included in the Supplementary information. All the sequence data in the Metabarcoting results section (SRR1300434 and MK032924) are also available in NCBI (https://www.ncbi.nlm.nih.gov/). All other data from this study are available in the main text or the supplementary materials.

Research involving human participants, their data, or biological material

Policy information about studies with human participants or human data. See also policy information about sex, gender (identity/presentation), and sexual orientation and race, ethnicity and racism.

Reporting on sex and gender	The terms "sex" and "gender" are not used in this study.
Reporting on race, ethnicity, or other socially relevant groupings	We are not reporting on "race, ethnicity, or other socially relevant groupings" in our study.
Population characteristics	This study was not involving human participants, their data, or biological material.
Recruitment	This study was not involving human participants, their data, or biological material.
Ethics oversight	This study was not involving human participants, their data, or biological material.

Note that full information on the approval of the study protocol must also be provided in the manuscript.

Field-specific reporting

Please select the one below that is the best fit for your research. If you are not sure, read the appropriate sections before making your selection.

☐ Life sciences ☐ Behavioural & social sciences ☒ Ecological, evolutionary & environmental sciences

For a reference copy of the document with all sections, see nature.com/documents/nr-reporting-summary-flat.pdf

Ecological, evolutionary & environmental sciences study design

All studies must disclose on these points even when the disclosure is negative.

Study description	The intracellular phosphate content of several species of benthic foraminifera from diverse marine environments has been quantified. In addition, we did ultrastructural analyses and element mapping on foraminiferal cells using cryoSEM/EDS and TEM/EDS to localize the intracellular phosphorous storage. For further characterization, in which form the phosphorous is stored, we performed extraction experiments and characterized the extracted phosphorous compounds using 31P-NMR. To characterize possible metabolic pathways, we performed comparative genomics on previously published transcriptomes and genomes of Ammonia veneta, Ammonia confertitesta, Globobulimina sp. and Reticulomyxa filosa. In addition, we used the results for the species specific phosphate storage and literature data about abundances of living foraminifera to estimate budgets for foraminiferal phosphate storage in the Southern North Sea and the Peruvian OMZ. Those were compared with the riverine phosphorous runoff in those regions.
Research sample	Most of the samples are freshly sampled benthic foraminiferal specimens, which have been directly sampled from seafloor sediments during several research cruises. Only the specimens of Ammonia veneta, that have been used for some of the TEM/EDS and cryo-SEM/EDS and intracellular phosphate concentration analyses are from a lab culture, which is described in detail in the methods section. As stated above, transcriptomes and genomes for the comparative genomics were already published before and have been taken from databases. All details are provided in the methods section.
Sampling strategy	We were specifically targeting different environments with various ranges of redox conditions. Specimens from the freshly collected samples have been chosen according to the foraminiferal species, that were present in the samples. Sediment samples have been processed immediately to provide results as close to the foraminifera´s natural habitat as possible. From these samples we picked as many living foraminiferal specimens as possible and tried to cover all foraminifera species that were present within the sample. We

	aimed to sample triplicates for all foraminifera species that were analyzed for the intracellular phosphate storage. For some species that were rare, we only were able to analyze one sample or duplicates, due to the lack of a sufficient number of specimens. All this is stated in datail within our datatables. Each sample contained between 1-70 living foraminifera specimens, depending on the average size and phosphate content of the analyzed foraminifera species. All samples were monospecific.<br>For the NMR analyzes, we needed huge samples, containing ~1000 living specimens. We chose the species Ammonia conferitesta for these analyses, since this species is very abundant within the easily accessible intertidal mudflats of Friedrichskoog. In addition, this species has the highest intracellular phosphate content, which reduced the required sample size.
Data collection	Data about number of sampled specimens for intracelular phosphate quantification has been recorded by Nicolaas Glock. Data about the size of specimens has been acquired by measurements on microscope images and documented by Nicolaas Glock. Quantification of phosphate concentrations from the extracted samples have been measured using segmented flow injection analysis by Andre Mutzberg and Akiko Makabe who also did the data documentation. NMR-analysis and data documentation has been done by Thomas Hackl. TEM-EDS and cryo-SEM/EDS analyses and data documentation has been done by Satoshi Okada.
Timing and spatial scale	No timeline experiments have been performed for this study.
Data exclusions	No data was excluded from the analyses.
Reproducibility	As mentioned above: For the intracellular phosphate quantification, we aimed for triplicates for each foraminifera species. In some cases for rare speces, we only performed single analyses or duplicates. All this is documented in detail within our data tables. For the NMR-Data we used duplicates of samples, containing ~1000 specimens of Ammonia confertitesta and the spectra of both replicates were nearly the same as shown in the supplements. Another replicate of ~1500 specimens has been analyzed to measure the 31P-NMR spectrum directly within the living specimens. For TEM-EDS, we analyzed a total of 41 images on 3 different specimens of Ammonia venata. For the cryo-SEM/EDS we analyzed three different specimens of Bolivina spissa. All contained the P- and Ca-rich structures, described in the paper.
Randomization	Samples were divided by species level. Specimens from the newly collected samples have been chosen according to the foraminiferal species, that were present in the samples. From these samples we picked as many living foraminiferal specimens as possible and tried to cover all foraminifera species that were present within the sample.
Blinding	This study did not contain any randomized control trials, making blinding irrelevant.

Did the study involve field work? ☒ Yes ☐ No

Field work, collection and transport

Field conditions	If possible, sampling time during ship cruises was chosen during good weather with low waves and winds to provide optimal conditions for sampling undisturbed sediments, using a multicorer. Weather and air temperature are not relevant for this study, except maybe at the intertidal mudflats of Friedrichskoog, since samples were retreived from the seafloor. Weather for sampling at the intertidal mudflats off Friedrichskoog was rainy in both November 2021 and May 2023. Air temperature in Friedrichskoog was 19.8°C in May 22nd 2023 and 6.8°C in November 25th 2021.
Location	All documented in table 2 of the main paper.
Access & import/export	No genetic material has been im- or exported for this study. No large animals have been sampled. The studied organisms include only foraminifera (microscopic protists).<br><br>Regarding sampling in the intertidal mudflats of Friedrichskoog during November 2021 and May 2023: Samples have been retrieved in compliance with state and national laws from a publically accesible non-protected spot in the German Wadden Sea at the town of Friedrichskoog. Sampling at Friedrichskoog was organized by the University of Hamburg.<br><br>Regarding to core retrievel during the RV Meteor cruise M176/2: Core retrieval was carried out exclusively outside the EEZ in compliance with international law and regulations of the neighbouring countries as mediated by the "Control Station German Research Vessels" of the University of Hamburg. Sampling cruise was organized by GEOMAR.<br><br>Samples during the Japanese cruises (R/V Kaimei cruise in September 2019, R/V Yokosuka cruise in May 2022, field trip to Hirakata Bay, Yokohama (Japan) in 2015) were collected from non-protected areas in Japanese territory or the Japanese EEZ in compliance with Japanese and international law. Sampling campaigns were organized by JAMSTEC.<br><br>Samples from the Bedford Basin (March 2022) were collected from non-protected areas in Canadian territory in compliance with Canadian and international law. The cruise was organized by Dalhousie University in Halifax.
Disturbance	The main disturbance by this study is related to the fact that foraminifera specimens had to be extracted from the sediments before the analyses. We aimed to minimize this disturbance by immediate sample processing and preparation after sampling. Only a few samples from the Bedford Basin had to be stored for 1-2 days in the cooling room, before it was possible to process them, due to the lack of time during the field trip.

Reporting for specific materials, systems and methods

We require information from authors about some types of materials, experimental systems and methods used in many studies. Here, indicate whether each material, system or method listed is relevant to your study. If you are not sure if a list item applies to your research, read the appropriate section before selecting a response.

Materials & experimental systems

n/a	Involved in the study
☒	☐ Antibodies
☒	☐ Eukaryotic cell lines
☒	☐ Palaeontology and archaeology
☒	☐ Animals and other organisms
☒	☐ Clinical data
☒	☐ Dual use research of concern
☒	☐ Plants

Methods

n/a	Involved in the study
☒	☐ ChIP-seq
☒	☐ Flow cytometry
☒	☐ MRI-based neuroimaging

Plants

Seed stocks	No plants were involved in this research.
Novel plant genotypes	No plants were involved in this research.
Authentication	No plants were involved in this research.
